# Ecotoxicological health risk analysis of different fish species from the Black Sea coast of Türkiye

**DOI:** 10.1038/s41598-025-87875-y

**Published:** 2025-08-22

**Authors:** Evrim Sibel ÖNEL, Mustafa TÜRKMEN, Erkan KALIPCI

**Affiliations:** 1 Faculty of Science, Department of Biology, Giresun University, Giresun city, Turkey; 2 Faculty of Engineering, Deparment of Geomatics Engineering, Giresun University, Giresun city, Turkey

**Keywords:** MPI, HI, THQ, target cancer risk, fish, Black Sea, Environmental impact, Metals

## Abstract

The aim of this study, fourteen potential toxic elements (PTE) levels were measured in seven different fish consumed intensively in eight provinces along the Black Sea coastline of Türkiye. Furthermore, human health risk assessment from fish consumption was investigated using multiple statistical approaches, and a spatial distribution map of PTEs in the region was prepared. The average PTEs levels in the seven fish muscle are listed in the following order: Fe > Zn > As > Cu > Mn > Ni > Co > Hg > Cd > Pb > Cr with the values of 17.028 > 14.288 > 2.045 > 1.017 > 0.721 > 0.064 > 0.032 > 0.031 > 0.027 > 0.021 > 0.018 µg g-1, respectively. In the seven fish species examined, the sum of the mean values of toxic metals and essential metals was lowest in MM (18.75 µg g-1) and highest in EE (58.16 µg g-1) and MB (57.66 µg g-1 ). The maximum values of PTEs measured in fish were mostly observed in the provinces of Kastamonu, Sinop, Artvin and Rize. For all PTEs, hazard index (HI), target hazard quotients (THQ) and metal pollution index (MPI) from metal intake by ingesting seven fish species were less than 1, indicating no risk from consumption. It can be said that there is unlikely to be a risk of potential cancer issues in those who consume the fish used in this study, as the estimated daily PTEs intakes in fish were found to be much lower than acceptable daily intake levels. It is recommended to introduce biomonitoring species to these areas and to take preventive measures against metal pollution in order to monitor metal pollution from industrial and discharge areas, to identify, monitor and compare heavy metal sources.

**Supplementary Information:**

The online version contains supplementary material available at 10.1038/s41598-025-87875-y.

## Introduction

Today, potentially toxic elements (PTEs) have become the most important global environmental concern as they pose ecological and human health risks due to their natural persistence, non-biodegradability and cumulative nature in aquatic ecosystems^1^. Coastal systems are heavily exposed to PTEs pollution from maritime, agricultural, urban and industrial activities. In developing countries, metal pollution is critical due to handicraft and organized mining, lack of control over rural and urban wastes, lack of adequate treatment for wastewater sent directly to natural water systems^2,3^. In particular, the number of scientific studies showing that industrial wastewater has negative ecological effects and toxic effects on the environment and living organisms in the environment, the immune system and different tissues, respectively, is increasing day by day^4–7^. Today, PTEs are major pollutants of the environment and have become a fast-growing problem for water systems such as oceans, lakes and rivers in areas where industry is concentrated^8,9^. PTEs are stored in water, sediment and aquatic organisms because they do not degrade for long periods of time. Potentially toxic elements (PTEs), such as heavy metals, remain active for a long time as a result of accumulation in the body and participation in the food chain, and when they exceed the tolerance limit, they have a toxic effect^10,11^. Environmentally persistent PTEs can bioaccumulate in marine products and the food chain through respiration, adsorption and ingestion. Therefore, PTEs have high toxicity to all living organisms and cause significant health problems because they are not biodegradable^12–15^.

In aquatic environments, degradation-resistant PTEs accumulate in sediments over time, increasing the pollution level of the aquatic environment^16,17^. These harmful PTEs can be absorbed through the skin surfaces, especially of fish species and other marine organisms, and accumulate in soft tissues^1^. Fish has become one of the most widely consumed seafood products due to its high protein and unsaturated omega fatty acids^18–21^. The highest aquaculture consumption rate was determined in Asia, with 72% in China, Indonesia, India and Japan. Fish consumption per capita is 5.4 kg in low-income countries with food deficits, 26.5 kg in high-income countries, while the world average is 22 kg. in Türkiye, fish consumption per capita is 6.3 kg. After consuming these fish species, toxic pollutants enter the human body through the bioaccumulation process and can cause serious health problems^22–25^. Depending on dietary habits, the abundant consumption of seafood, especially for those living along the coastline, can lead to potential public health risks^26–31^. The exposure of fish, which are a source of protein, to PTEs leads to the development of toxic effects in the fish. The consumption of these fish by humans can lead to reproductive and hematological effects in adults and children, as well as issues such as cardiovascular diseases, developmental abnormalities, nervous system disorders, kidney and liver damage, and memory loss^12,32,33^. Additionally, the PTEs accumulated in fish are carcinogenic to humans and animals, and it has been reported that they cause skin discoloration and infertility in the reproductive system^34–36^. Children are very susceptible to PTEs poisoning due to their growth and metabolism. PTEs poisoning negatively affects the nervous system, mental development and behavior in children aged 2–4 years^37^. For children, potentially toxic elements such as As, Hg, Pb and Cd have been linked to autism, mental retardation, attention deficit disorder and death^38^. In the Black Sea coastal strip, chemical pollutants such as oil, heavy metals, and pesticides are largely harming to the ecosystem and indirectly threatening the health of adults and children. PTEs in the aquatic environment disrupt the natural balance of the ecosystem by entering the tissue and cell structures of fish and causing great damage. They also cause bioaccumulation and toxicity of PTEs in humans through fish consumption; fish are vital in the food chain as they are an important source of protein^39,40^. The Black Sea, which was selected as the research area in this study, accounts for 76% of Türkiye’s fish production^41^. Therefore, in this study, a total of eight provinces, namely Artvin (ART), Rize (RZE), Trabzon (TRB), Giresun (GRS) in the Eastern Black Sea Region, Ordu (ORD) and Samsun (SMS) in the Central Black Sea Region, Sinop (SNP) and Kastamonu (KST) in the Western Black Sea Region, where fisheries along the Black Sea coast are intensive, were determined as the research area. The aims of this study are;

(i) To determine the levels of PTEs (Al, Cu, B, Mn, As, Fe, Se, Co, Cr, Ni, Hg, Zn, Cd and Pb) in fish, which are widely consumed in the human diet in Türkiye and other European countries, (ii) To assess the potential health risks of exposure to PTEs when fish is consumed by adults using multi-approach risk models (MPI, THQ, Estimated Daily Intakes (EDI) and HI], (iii) To prepare PTE accumulation map showing the spatial distribution of PTEs in fish along the Black Sea coastline by geographic information systems (GIS).

## Materials and methods

### Study area and fish collection

**Fig. 1:**
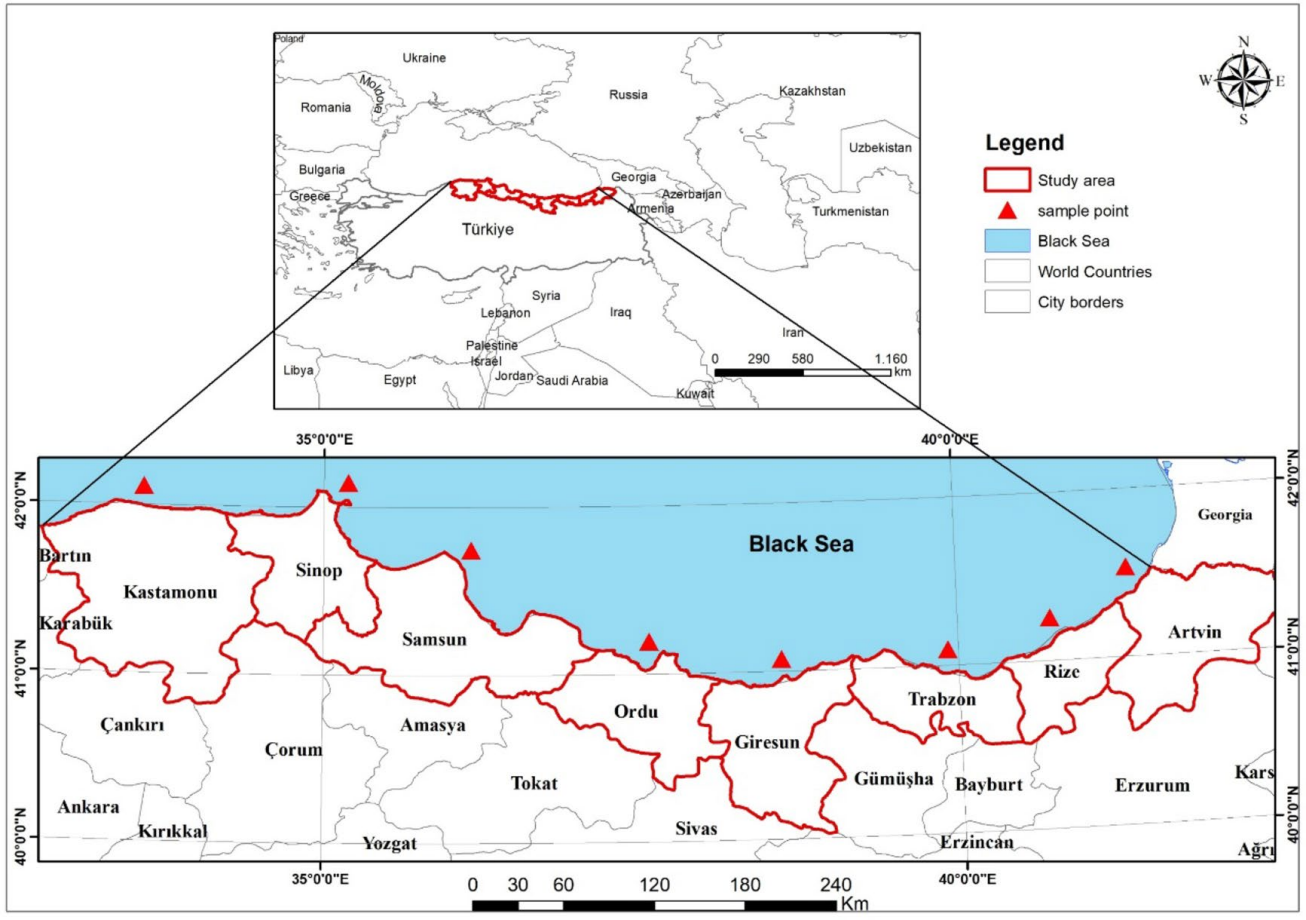
Sampling stations and study area.

The Black Sea, which accounts for more than half of fish production in Türkiye, has been chosen as the area of study along the Türkiye coastline. The Black Sea coastline is surrounded by six countries and our study area is located between 42.08 and 42.50 N latitude and 32.71–39.90 E longitude. The salinity of the Black Sea, which accounts for 76% of Türkiye’s fish production, is around 18% on the coastline, 23% on the surface and 36% at depth. In addition to the Sakarya, Kızılırmak and Yeşilırmak rivers, the Çoruh, Dnieper, Dniester and Danube rivers also flow into the Black Sea^42,43^. In the Black Sea Region, which was selected as the study area, fish samples were collected randomly from fishermen in a total of 8 provinces in Artvin (ART), Rize (RZE), Trabzon (TRB), Giresun (GRS), Ordu (ORD), Samsun (SMS), Sinop (SNP) and Kastamonu (KST) in the Black Sea coastal waters of Türkiye (Fig. 1). *Pomatomus saltatrix* (PS), *Mullus barbatus* (MB), *Trachurus trachurus* (TT), *Engraulis encrasicolus* (EE), *Mugil cephalus* (MC), *Merlangius merlangus* (MM) and *Sarda sarda* (SS), which are widely consumed throughout Türkiye and have economic value, were sampled in September and October 2021. The fish were caught with the help of a local fishing boat. In order to provide objective and reliable data, 5 samples each for *Mugil cephalus* and *Sarda sarda* and 10 samples each for the other fish species were collected at all stations and studies were carried out with a total of 480 fish for 7 species and 8 provinces. The mean weight and length of seven different fish species collected from eight cities are given in Table 1.

**Table 1:** The fish species, length and weight information used in the study.

Scientific and local name	Total sample counts	Sample type	Average length and wight	ART	RZE	TRB	CITIES GRS	ORD	SMS	SNP	KST	Overall average
1.*Mullus barbatus (MB)* Barbun	**80**	Flesh (muscle)	**AL(cm)**	13.2 ± 1.3	12.5 ± 0.5	*10.8* ± 0.7	13 ± 1	**13.3** ± 1.2	12 ± 1	13 ± 0.5	13 ± 0.3	12.60
			**AW(g)**	19.5 ± 0.51	17.23 ± 0.54	*14.40* ± 0.92	17.67 ± 1.21	22.28 ± 1.55	17.86 ± 1.13	22.52 ± 2.01	**24.56** ± 2.24	19.50
2.*Pomatomus saltatrix* (PS) Çinekop	**80**	Flesh (muscle)	**AL(cm)**	15.8 ± 1.8	*14.5* ± 0.5	17 ± 2	15 ± 1	15.5 ± 1.5	16.5 ± 1.5	17.5 ± 2.5	**19** ± 2	16.35
			**AW(g)**	40.38 ± 1.43	*33.68* ± 1.16	48.70 ± 1.51	36.60 ± 3.96	48.74 ± 1.2	50.10 ± 2.08	62.40 ± 3.9	**79.58** ± 4.59	50.02
3.*Engraulis encrasicolus* (EE) Hamsi	**80**	Flesh (muscle)	**AL(cm)**	11 ± 0.5	10.5 ± 0.5	11 ± 1	*10.3* ± 0.7	10.8 ± 0.3	11.8 ± 0.7	**12.3** ± 0.2	11.3 ± 0.4	11.13
			**AW(g)**	8.28 ± 0.9	7.85 ± 0.9	8.27 ± 0.5	*6.84* ± 0.45	7.31 ± 0.4	8.87 ± 1	**9.42** ± 1.2	8.47 ± 1	8.16
4.*Trachurus trachurus* (TT) İstavrit	**80**	Flesh (muscle)	**AL(cm)**	*11* ± 0.7	*11* ± 0.9	12 ± 0.9	*11* ± 1	12 ± 0.8	*11* ± 0.7	**13** ± 0.6	*11* ± 0.9	11.50
			**AW(g)**	13.37 ± 1.7	13.62 ± 0.7	15.54 ± 2	12.86 ± 0.6	14.21 ± 0.5	*10.99* ± 1.3	**25.51** ± 0.4	13.65 ± 0.8	14.97
5.*Mugil cephalus* (MC) Kefal	**40**	Flesh (muscle)	**AL(cm)**	*22* ± 2	24 ± 1	28.5 ± 5	23 ± 1	32 ± 6	24 ± 3	**34** ± 5	24 ± 2	26.44
			**AW(g)**	*89.92* ± 8.3	128.21 ± 10.1	263.67 ± 10.4	125.68 ± 4	298.74 ± 14.9	106.08 ± 6.7	**340.11** ± 22.9	137.04 ± 7.4	186.18
6.*Merlangius merlangus* (MM) Mezgit	**80**	Flesh (muscle)	**AL(cm)**	15.5 ± 0.5	16.3 ± 0.8	16 ± 1	*13.3* ± 0.8	16.5 ± 0.5	16.5 ± 0.9	**18.3** ± 0.3	16 ± 0.5	16.05
			**AW(g)**	30.28 ± 1.1	27.66 ± 2.5	31.00 ± 0.9	*18.87* ± 2.6	30.13 ± 3.6	28.54 ± 2.9	**34.03** ± 3.6	30.36 ± 1.7	28.86
7.*Sarda sarda* (SS) Palamut	**40**	Flesh (muscle)	**AL(cm)**	35 ± 2.9	35.5 ± 2.7	33 ± 1.9	36 ± 3.2	*30* ± 2.9	36 ± 3.9	**38** ± 4.2	35 ± 2.1	34.81
			**AW(g)**	653.9 ± 58	505.8 ± 33	416.1 ± 31	559.9 ± 21	*285.5* ± 32	605.4 ± 67	**710.0** ± 86	573.4 ± 35	538.75

*ART* artvin province, *RZE* rize province, *TRB* trabzon province, *GRS* giresun province, *ORD* ordu province, *SMS* samsun province, *SNP* sinop province, *KST* kastamonu province. *Underlined values ​​indicateminimumdata,

* bold values ​​indicate maximum data.*

### Preparation of samples for metal analysis in fish

**Fig. 2:**
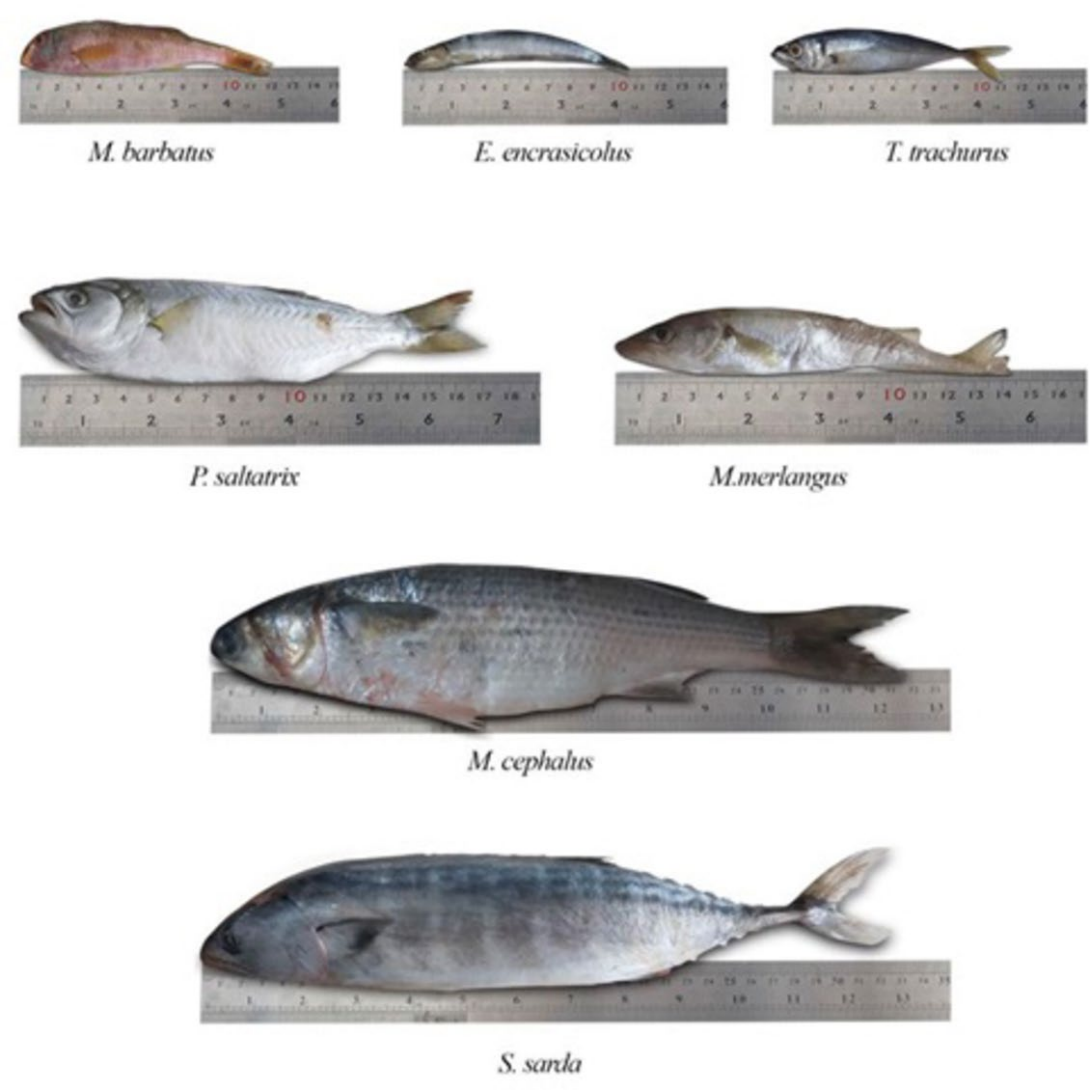
Seven different fish species abundantly consumed in the Black Sea used in this study.

After the collection of fish from eight different stations in the Black Sea coastal waters of Türkiye (Fig. 2), the total weight and length of the specimens brought to the laboratory on ice were measured to the nearest millimeter and gram before dissection (Table 1). Muscle tissue (5.0 g) taken from fish samples was labeled, placed in bags, and stored at -18 °C until analysis^44^. Fish species collected for the study (up to 1.5 g) were digested in Teflon containers (HPR-FO-67) containing a concentrated mixture of HNO_3_ (suprapur 65%) and H_2_O_2_ (suprapur 30%) (7:1) according to temperature and this process was performed using a microwave digestion system (Milestone SK10). The acid was heated at 200 °C for 15 min after the addition of Teflon and remained at the same temperature for 15 min. The digested solution was transferred to 50 ml polypropylene tubes and filled to 50 ml with ultrapure water. For filtering, 0.45 m filter paper was employed^45^. The concentrations of the elements were measured by Inductively Coupled Plasma Mass Spectrophotometer (ICP-MS, Agilent 7700X). Quality assurance and control was performed using triple measurement and certified reference material (UME CRM 1201 spring water, Lobster TORT-2, BCR-185r liver, SEM 2016). Standard solutions provided by Agilent (27 element mixture: 8500 − 6940 2 A and 8500 − 6940 Hg) were used for calibration curves. Analytical precision was within ± 10%. In this study, 1ppm internal standard (Agilent 5188–6525) was analyzed continuously with samples. The concentrations of PTEs in fish tissues were calculated in terms of wet weight (ww) as µg g^− 1^.

### Analysis of health risk indexes

The effects of consumed fish on public health were investigated using the determined PTEs concentrations in fish and multifaceted health risk indices; Metal pollution index (MPI), Target hazard quotients (THQ), Hazard index (HI), Estimation of weekly intake rate (EWI), and Estimation of daily intake rate (EDI). Average potential toxic elements values and index formulas used in the calculations are given in the Supplementary Material (see the Supplementary Material File).

### Statistical analysis

Statistical analysis of the results was performed using IBM SPSS Statistics 22 package program. Anova one-way analysis of variance was performed for variables with normal distribution in intergroup comparisons. Duncan test was used to determine the differences during ANOVA. *p* < 0.05 value was accepted as statistically significant. Pearson’s correlation analysis (PCC) and Two-way cluster analysis was applied to reveal the relationship between heavy metals. The accumulation thematic spatial analysis map of PTEs in fish was created using ArcGIS 10.8 software.

### Compliance with ethical standards

In this study, the authors declare that there is no conflict of interest between them. Autopsy; The fish used in this study were not subjected to torture in any way, as the autopsy was carried out after the fish died naturally. All procedures performed in the studies were performed in accordance with the ethical standards of the institutional and/or National Research Committee and the 1964 Helsinki declaration and ARRIVE guidelines and its later amendments or comparable ethical standards. We confirm that all methods were carried out in accordance with the relevant guidelines and regulations.

## Results and discussion

### PTEs value levels detected in fish

**Fig. 3:**
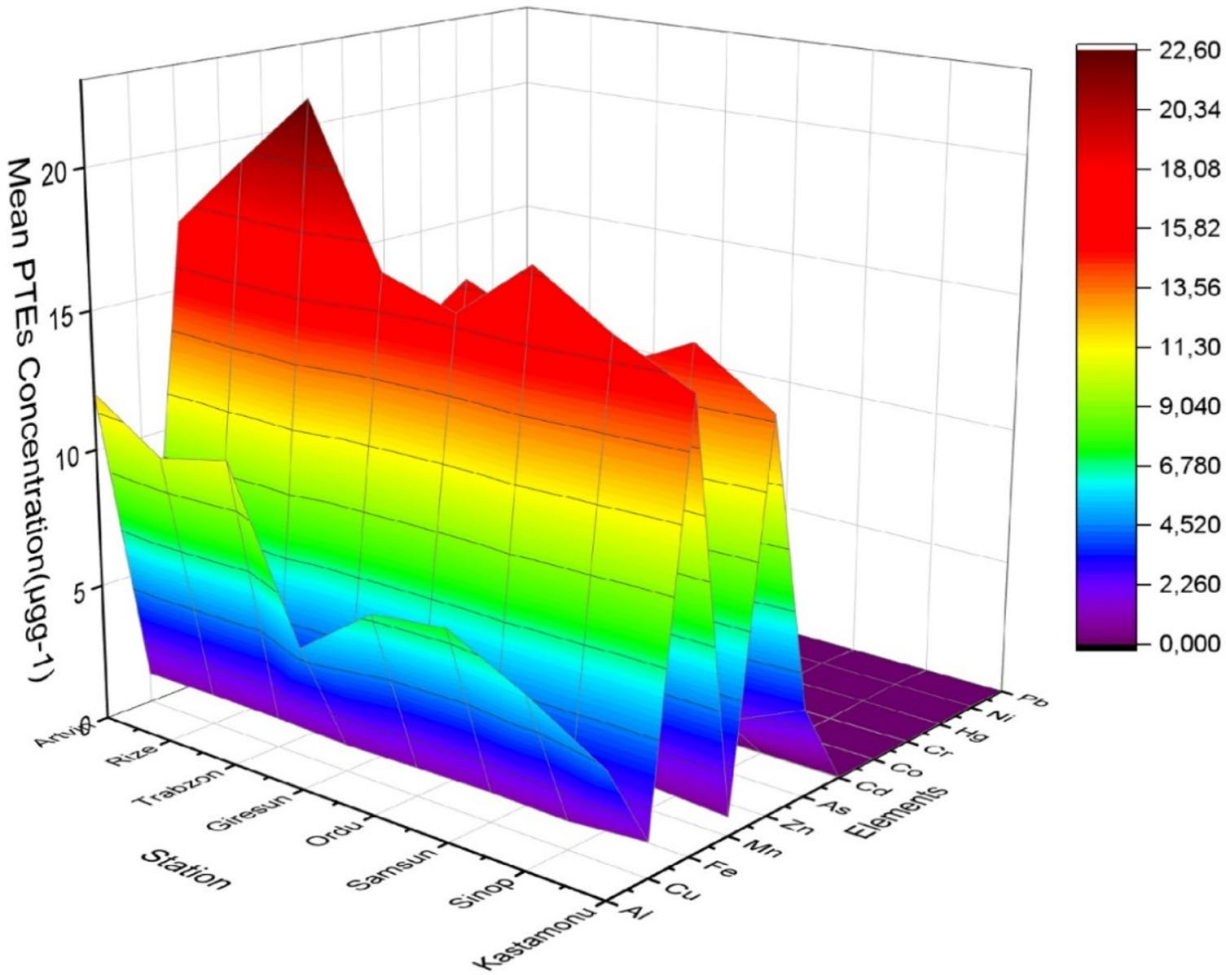
The distribution of combined mean PTEs in the fish species according to sampling stations.

Mean PTEs concentrations in, *Pomatomus saltatrix* (PS), *Trachurus trachurus* (TT), *Mullus barbatus* (MB), *Engraulis encrasicolus* (EE), *Mugil cephalus* (MC), *Merlangius merlangus* (MM) and *Sarda sarda* (SS) fish species caught from 8 provinces on the Black Sea coastline of Türkiye, their maximum permissible limits (MPLs) and comparison with fish in other studies are given in Table SM1- Table SM7 (see the Supplementary Material File). The combined average PTE distribution of fish species according to sampling stations are shown in Fig. 3. In a total of 480 fish samples examined in the study, the highest average PTE levels were generally measured for Al, Fe, Cu, Zn, As and Se. The lowest levels were found for Co, Cd, Pb, Hg and Cr. Different researchers have also reported similar results in their studies^12,45–48^.

**Fig. 4:**
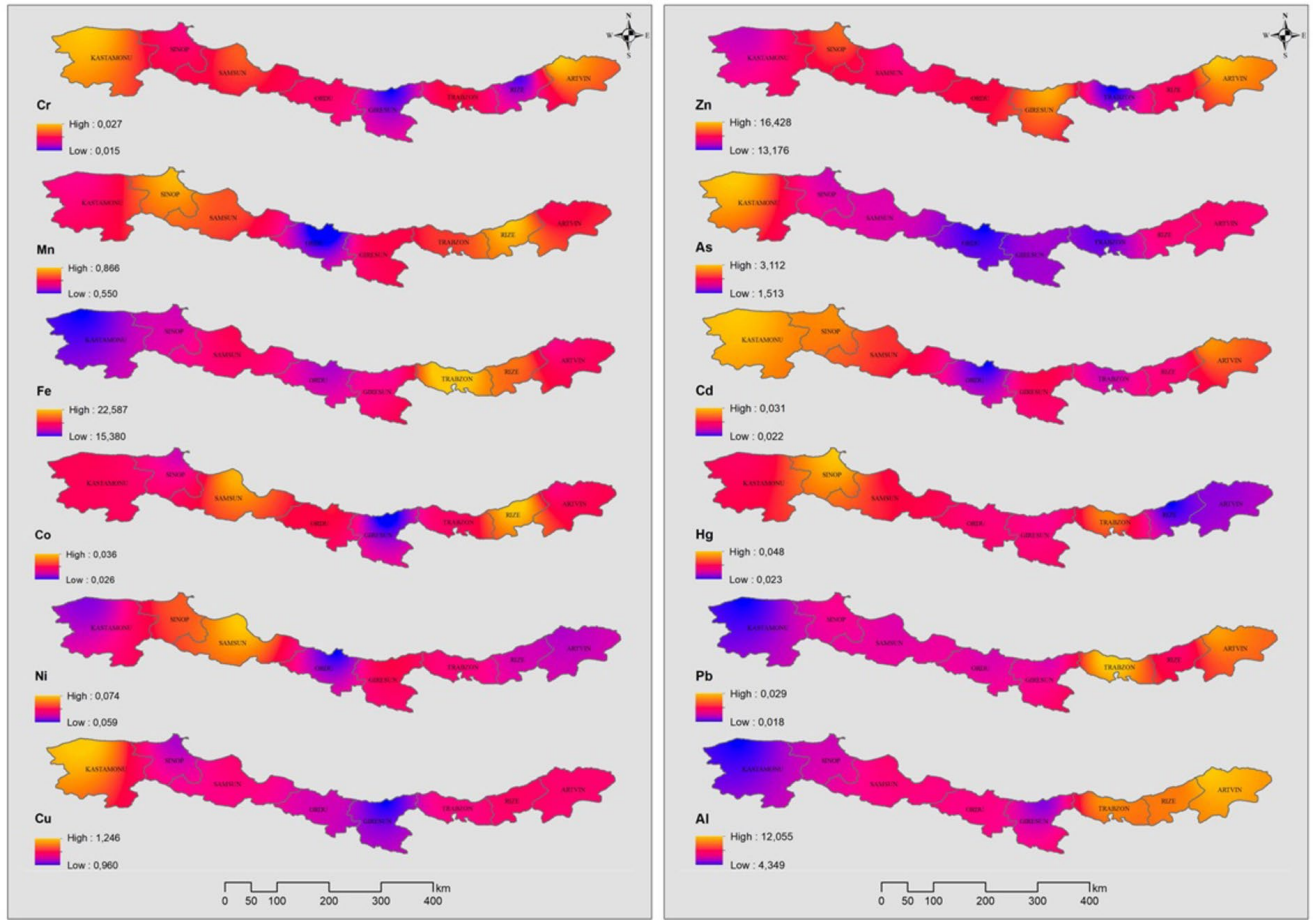
The thematic spatial analysis map of PTEs bioaccumulation in seven fish.

Thematic spatial analysis map showing the accumulation of PTEs (As, Fe, Cr, Co, Mn, Cu, Ni, Hg, Pb, Al, Zn and Cd) in fish from the Black Sea coastline is given in Fig. 4. In this study, a continuous surface was created for the study area by using the inverse distance weighted (IDW) approach, one of the interpolation techniques, with ArcGIS 10.8 software for thematic spatial distribution analysis. This method used in our study is based on the assumption that the spatially distributed sampling points in the study area are interconnected^49,50^. As seen in Fig. 4, the highest metal levels in fish sampled from Kastamonu province are Cr, Cu, As and Cd. The probable reason for these results is thought to be the mineral transportation in the port of İnebolu and the mixing of inland water with the sea in this interval, as well as shipyard activities^51^. The highest metal concentrations in fish sampled from Samsun province were Ni and Co. This is thought to be due to the Kızılırmak River, the third longest river in Turkey, which flows into the sea from the Bafra district of Samsun province^9,13^. The highest metal concentrations in fish sampled from Rize province were Mn, Co and Al. It is believed that this situation is caused by mining activities in the Rize province. Additionally, it is believed that the floods, waterlogging, and landslides caused by the heavy rainfall that occurred in the Western Black Sea Region before the fish sampling in August 2021 have increased metal pollution, particularly affecting the provinces of Kastamonu and Sinop. These results are similar to the previous study reported by Kalıpcı et al.^30^.

#### (Zn)

Assessing trace element levels and potential health risks in fish is critical today to ensure food safety and protect public health^52^. Therefore, the maximum acceptable level for zinc in edible fish is 100 µg g^− 1^ ww, as suggested by the WHO^53^ guidelines. In this study, Zn levels ranged from a average of 32.11 ± 7.45 µg g^− 1^ ww (Table SM1- Table SM7, see the Supplementary Material File). In addition, significant differences in Zn levels were observed among fish species, with EE having significantly higher levels compared to SS, MC, and MM (*p* < 0.05). In the current study, mean zinc levels in the fish species were lower than the previous studies^44,45^ but higher than the reported studys^30,54^. The average Zn levels in all fish species were below MPLs set by WHO^55^.

#### (As)

Similar to Zn, MB (5.31 µg g^− 1^) and EE (2.35 µg g^− 1^) had the highest mean concentrations of Arsenic. The mean arsenic the amounts in the other fish species were less than 1.80 µg g^− 1^. The lowest average levels of As were found in MC (0.99 µg g^− 1^) and PS (1.06 µg g^− 1^). Seven different fish species’ variations in arsenic content revealed that MB had a higher arsenic content than other species (*p* < 0.05). Arsenic levels in this study are lower than those in the earlier study by Ustaoğlu and Yüksel^52^ and Varol et al.,^56^ with Martinez-Gomez et al.,^57^. Copat et al.,^58^ reported a comparable average level for As for E. anchovy (5.28 µg g^− 1^) from the Mediterranean Sea in Italy (Table 2). The average As levels in muscles of all fish species were determined to be higher than the threshold value of reported by the Iranian Standard National Organization^59^ (0.60 µg g^− 1^) and Chinese Health Ministry^60^ (0.10 µg g^− 1^). These findings denote the critical importance of continuous monitoring and that it is necessary to raise public awarenessmoderate the As exposure risks tied to fish consumption.

#### (Cr)

Cr mean concentrations measured in muscle of MB, PS, EE, TT, MC, MM and SS, differed 0.04, 0.01, 0.01, 0.01, 0.04, 0.01 and 0.01 µg g^− 1^ (wet weights), in turn. The highest mean levels of Cr was found in MB, MC, (0.04 µg g^− 1^) and the lowest in PS, EE, TT, MM, SS (0.01 µg g^− 1^) in fish tissues. The average chromium levels for MB and MC differ significantly between other fish species (*p* < 0.05). Compared to this study, higher average Cr level in MB and MC (0.09 and 0.07 µg g^− 1^) was reported by Türkmen and Akaydın^61^. In our study, the average Cr levels found in all fish species were lower than the values found in the studies conducted by other researchers (Table SM1- Table SM5, see the Supplementary Material File). The average Cr levels in MB samples collected from the western Black Sea coastline by Kalıpcı et al.^30^ are compatible with the results of our study. The average Cr levels in muscles of all fish species were determined to be lower than the threshold value of reported by the Chinese Health Ministry (MHPRC)^60^ (2.0 µg g^− 1^) and World Health Organisation^53^ (0.01 µg g^− 1^).

#### (Fe)

Iron bioaccumulation was higher than all other PTEs in all sampled fish species with the exception of PS, MM, TT and EE when the bioaccumulation amounts of PTEs in the muscle is evaluated. The highest Fe average level was detected in the muscle of MB (37.96 µg g^− 1^ ww), and followed by muscle of EE (19.64 µg g^− 1^ ww). The lowest Fe mean level was detected in the muscle of MM (7.46 µg g^− 1^ ww). Bat et al.,^54^ reported lower average level of Fe for MB (2.3 µg g^− 1^) from the Black Sea coastline in Türkiye (Table 2). Iron levels were recorded between 68.6 and 163 µg g^− 1^ in the muscles of the fish caught from the Black Sea coastline and the Aegean Sea^62^. According to these results, the mean Fe concentration values ​​in our study were found lower. The mean Iron levels in seven fish species were below maximum permissible limits set by WHO^55^.

**Table 2:** Results of studies in the literature.

Species of fish	Sites	Al	As	Cd	Co	Cr	Cu	Fe	Mn	Ni	Pb	Zn	Reference
*P. saltatrix*	Black Sea, Türkiye	-	0.99	0.015	0.008	0.035	0.72	8.81	0.27	0.10	0.022	11.11	^30^
*E. encrasicolus*	Black Sea, Türkiye	-	-	0.09	0.03	0.55	0.55	-	0.59	1.62	0.19	16.9	^12^
*M. barbatus*	Black Sea, Türkiye	< 0.5	1.30	< 0.02	-	-	< 0.5	2.3	-	-	< 0.05	3.2	^54^
*M. merlangus*	Black Sea, Türkiye	< 0.5	1.24	< 0.02	-	< 0.1	< 0.5	0.87	0.93	0.01	< 0.05	3.4	^54^
*E. anchovy*	Black Sea, Türkiye	-	0.25	0.27	-	1.12	1.96	75.7	9.1	1.93	0.3	38.8	^44^
*E. anchovy*	Mediterranean Sea, Italy	-	5.28	0.001	-	0.009	-	-	0.257	0.046	0.005	6.58	^58^
*M. horse mackerel*	Black Sea, Türkiye	-	-	0.3	0.17	0.5	1.68	57.6	1.92	0.62	1.31	8.15	^45^
*P. saltatrix*	Black Sea, Bulgaria	-	0.77	0.008	-	-	0.8	-	-	0.009	0.03	10.0	^46^
*M. merlangus*	Black Sea, Türkiye	-	0.17	0.21	-	0.12	1.32	98.1	0.16	0.135	0.53	65.4	^44^
*S. sarda*	Black Sea, Bulgaria	-	0.410	0.015	-	-	0.660	-	-	0.11	0.06	10.0	^46^
*T. mediterraneus*	Black Sea, Türkiye	-	-	0.03	-	-	16.7	57.2	-	-	0.63	42.6	^45^
*Red mullet*	Mediterranean Sea, Spain	-	19.8	0.0011	-	-	0.35	-	-	-	0.005	3.65	^57^
*P. saltatrix*	Black Sea, Bulgaria	-	0.77	0.008	-	0.06	0.8	5.0	-	0.009	0.03	10.0	^63^

#### (Mn)

In this study investigation, the muscle tissues of EE had the greatest value of Mn average content (2.15 µg g^− 1^), whereas that of MB had the closest mean values (1.39 µg g^− 1^). The lowest mean levels of Mn were 0.18 µg g^− 1^ in the muscle tissues of SS. The mean concentrations of manganese in EE muscle caught in the Black Sea coastline were investigated and determined as 0.59–1.13, 0.70–2.82 and 1.06–3.24 (µg g^− 1^ ww), respectively^12,45,48^. The Mn values of EE in the current study were found in the range of 1.02 to 3.44 (µg g^− 1^ ww) in the wet muscle, which was found to be high compared to previous studies. The mean manganese levels in muscles of EE (2.15 µg g^− 1^ ww) and MB (1.39 µg g^− 1^ ww) seven fish species were calculated to be higher than the threshold value of 1 µg g^− 1^ reported by the World Health Organisation^53^.

#### (Ni)

In this study, the highest mean nickel level was detected in the muscle of MB (0.13 µg g^− 1^ ww), and followed by muscle of EE (0.12 µg g^− 1^ ww) and MC (0.07 µg g^− 1^ ww). Between seven fish species, there were substantially differing mean Ni contents (p 0.05). Türkmen and Akaydın^61^ with Varol et al.,^56^ reported higher average level of Ni for MB (2.54 µg g^− 1^; 0.25 µg g^− 1^ ) from the Black Sea coastline in Türkiye, respectively. All fish species’ mean Ni levels were found to be below the threshold of 0.5–1 µg g^− 1^ reported by the World Health Organisation^55^.

#### (Cd)

Cd mean concentrations in fish muscle were found to be highest in EE (0.07 µg g^− 1^) and the lowest in PS (0.01 µg g^− 1^) and SS (0.01 µg g^− 1^). Our study has a lower average Cd level compared to previous studies^48,61,64^. There were differences in cadmium levels among fish species that were statistically significant (*p* < 0.05). Except for EE, all fish samples that were examined were determined to have Cd concentrations below the allowable limits by Turkish Food Codex^65^ and European Commission^66^.

#### (Co)

Cobalt average bioaccumulation level in the muscles of fish was found between 0.01 and 0.08 µg g^− 1^ ww. The highest Cobalt mean concentration was detected in the muscle of MB (0.08 µg g^− 1^ ww), and the closest level to this value was detected in the muscle of EE (0.05 µg g^− 1^ ww). Statistically significant differences were found in the levels of Co among fish species (*p* < 0.05). The average Co levels in the seven fish muscle are listed in the following order: MB > EE > MC > TT > MM > PS > SS. The Co average concentrations measured in our study were lower than the concentrations measured from the fish in the Black Sea coastline^30,45,67^.

#### (Pb)

**Fig. 5:**
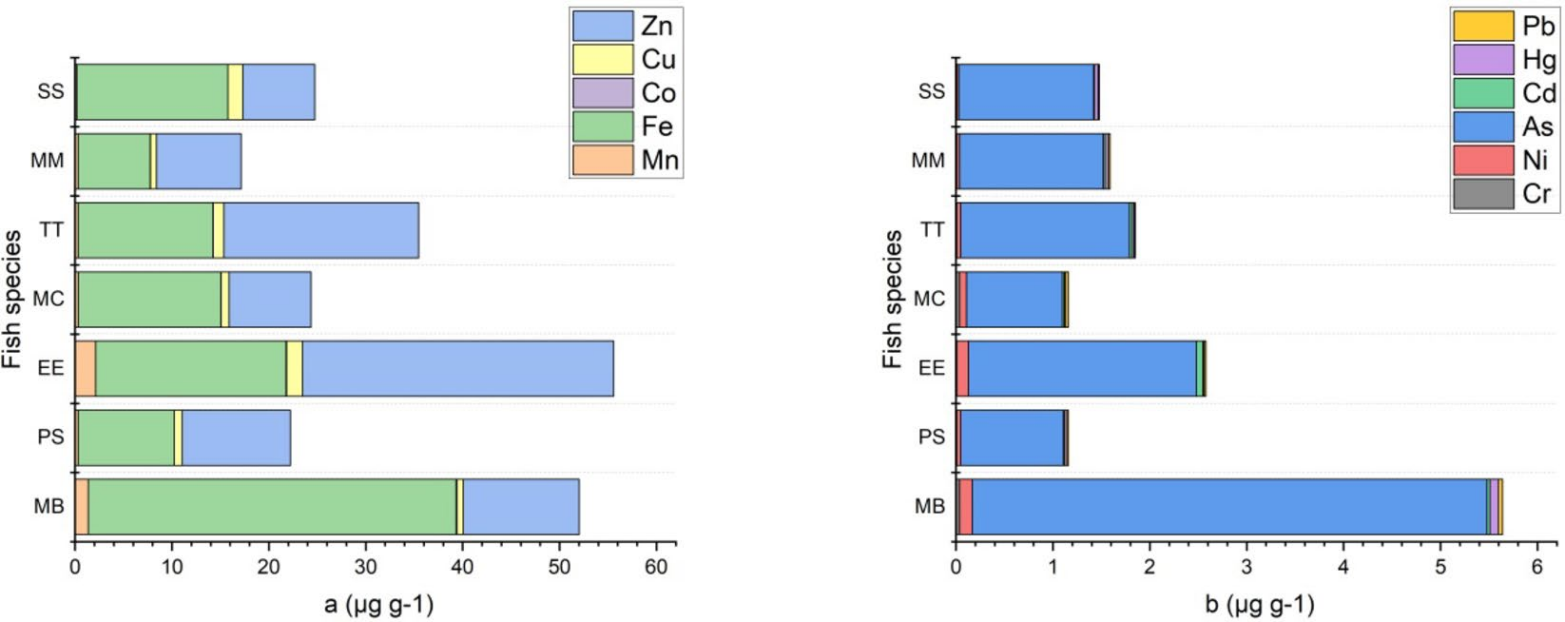
Mean concentration (as µg g^− 1^) of the distribution of essential metals (**a**) (Fe, Mn, Co, Zn and Cu) and toxic metals (**b**) (Cr, Pb, Ni, Cd, Hg, and As) in seven fish species.

Mean Pb concentrations were found to be 0.04, 0.03, 0.02, 0.02, 0.02, 0.01, and 0.01 µg g^− 1^for MB, MC, PS, MM, EE, TT and SS respectively (Fig. 5b). These values are less than what was previously reported^20,21,52,68,69^. The maximum lead levels permitted for fish are 2.0, 0.3, 0.5, 0.5 and 0.3 µg g^− 1^ according to the World Health Organisation^55^, Turkish Food Codex^65^, Chinese Health Ministry^60^, FAO^70^, and EC^66^ respectively. Seven fish samples tested for lead were found to have levels below the allowable limits.

#### (Cu)

The highest average copper levels were found in EE (1.63 µg g^− 1^ ww) and SS (1.51 µg g^− 1^ ww), while the average copper levels in the rest of species were below 1.12 µg g^− 1^ ww. Significant differences were found in the levels of Cu among fish species (*p* < 0.05). Türkmen and Öğütçü^12^ reported lower concentration of mean Cu for EE (0.55 µg g^− 1^ ww) from Black Sea in Türkiye. The mean Cu levels found in other fish species were lower than those reported in the literatüre. The mean copper levels in all fish species were far below MPLs (30 µg g^− 1^ ww) set by FAO^70^.

#### (Hg)

The highest average levels of mercury were found in MB (0.09 µg g^− 1^), PS (0.04 µg g^− 1^), and MM (0.03 µg g^− 1^) (Fig. 5b) respectively. Hg levels in this study are lower than those in the earlier study by Erdem et al.^47^. A similar average value of mercury was reported by Duyar and Bilgin^71^ reported that the mean the value of Hg was comparable for TT (0.02 µg g^− 1^ ww) in Black Sea coastline. According to Turkish Food Codex^65^, FAO^70^, and EC^66^, there should be no more than 0.5, 0.5, and 0.5 µg g^− 1^ of Hg in fish, in turn. Our this study, average Hg levels were determined to be within the allowed MPLs in seven fish samples.

### Arrange of bioaccumulating PTEs levels in seven fish species

**Fig. 6:**
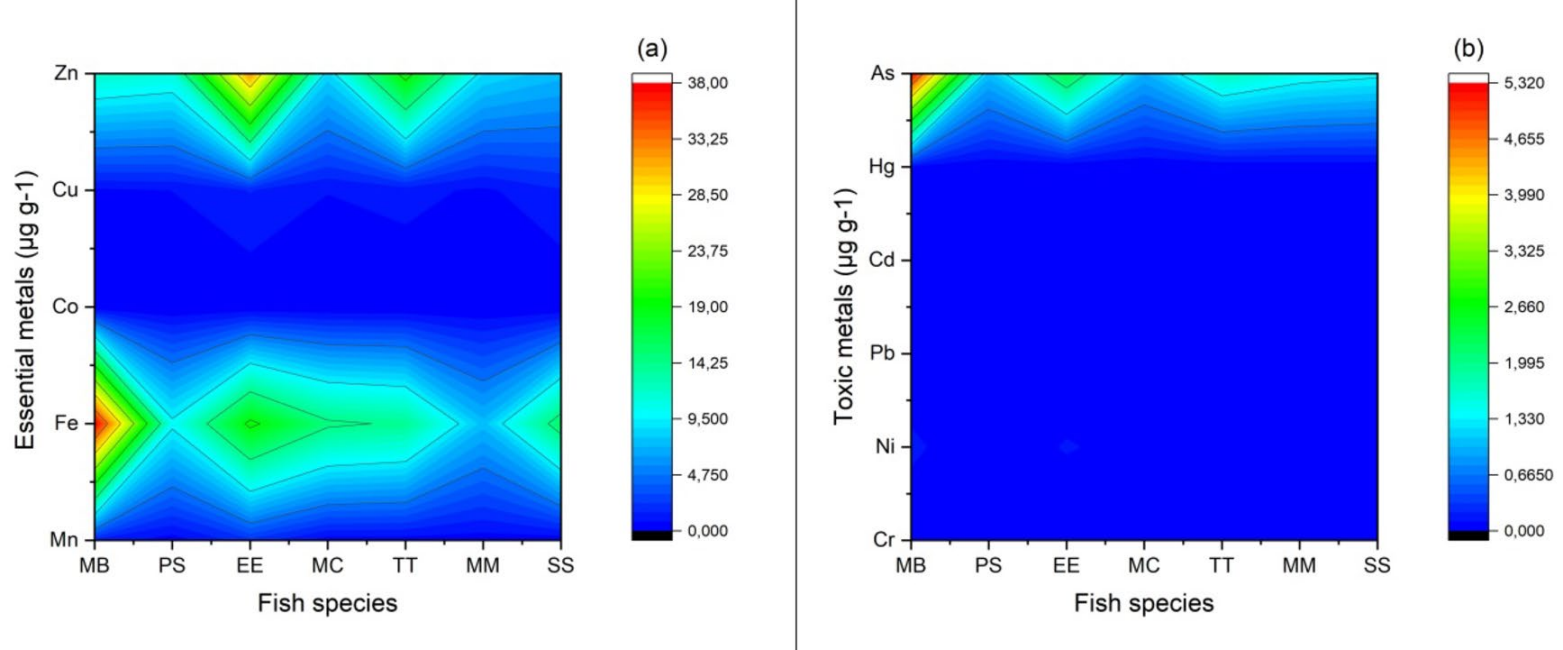
Average level (as µg g^− 1^) of the sum of essential metals (**a**) (Cu, Mn, Zn, Co, and Fe) and toxic metals (**b**) (Cr, Hg, Ni, Pb, Cd, and As) in seven fish species [*Pomatomus saltatrix* (PS), *Mullus barbatus* (MB), *Engraulis encrasicolus* (EE), *Mugil cephalus* (MC), *Trachurus trachurus* (TT), *Merlangius merlangus* (MM), *Sarda sarda* (SS)].

In this study, seven fish species were ranked according to the sum of the mean concentration levels of bioaccumulating essential metals (Fe, Mn, Zn, Cu and Co) and potentially toxic metals (As, Cr, Hg, Pb, Cd, Ni). In terms of bioaccumulation of base metal concentrations, EE had the highest and MM had the lowest concentrations (Fig. 6a). In terms of bioaccumulation of toxic metal concentration level, the highest level was detected in MB and the lowest level in PS and MC fish (Fig. 6b). The highest value of the sum of the mean bioaccumulated toxic metal and essential metal levels in all seven fish species was measured in EE (58.16 µg g^− 1^) and MB (57.66 µg g^− 1^) fish. The average concentration distributions of essential metals and potentially toxic metals are shown in Fig. 5. According to this, the highest essential metals Zn and the highest toxic metals As were measured in EE. In MB, the highest essential metals measured are Fe and the highest toxic metal is arsenic. (Fig. 5). The average Fe levels in the seven fish muscle are listed in the following order: MB (37.96) > EE (19.64) > SS (15.61) > MC (14.71) > TT (13.89) > PS (9.93) > MM (7.46) (µg g^− 1^). The average arsenic levels in the fish muscle tissues are listed in the following order: MB (5.31) > EE (2.35) > TT (1.74) > MM (1.48) > SS (1.39) > PS (1.06) > MC (0.99) (µg g^− 1^). The highest value of toxic metals and essential metals were measured in MB in seven fish species. It is thought that the level of potential toxic elements is high due to the fact that MB consumes mostly benthic invertebrates^18,30^.

### Health risk prediction of bioaccumulating PTEs in tissue of seven fish species

In previous studies, many researchers have reported that consuming fish with MPI values > 1 as food could pose a potential health risk^18,30^. As a result of the calculation made in our study; MPI quantitys in fish muscles were 0.685 in MB, 0.239 in PS, 0.457 in EE, 0.319 in TT, 0.322 in MC, 0.225 in SS and 0.273 in MM. Lower MPI levels were calculated in the muscle tissue of SS and MC fish with higher weights than MB and EE fish with lower weights, and these results were found to be consistent with previous studies^72,73^. Also, the average MPI quantity was calculated as 0.360 for seven fish in this study. In this study, the average MPI quantitys in the muscles of all fish species were lower than those reported in previous studies by Türkiye, Greece, Belgium, Spain, Italy, Portugal, and France, but higher than those reported by Kalıpcı et al.^30^ in the Black Sea coastline of Türkiye (Table 3). In the current study, the Metal Pollution Index (MPI) levels of seven fish species are < 1, and therefore, it is believed that they do not pose health risk concerns for adults and children.

**Table 3:** Comparison of MPI quantity in different countries. (adapted from Kalıpcı et al.^30^).

Country	MPI	References
Türkiye	0.36	This study
Portugal	2.38	^74^
China	0.29	^75^
İtaly	5.84	^76^
Türkiye	1.33	^18^
Greece	1.87	^77^
Türkiye (Black Sea)	0.27	^30^
France	0.67	^78^
Spain	2.11	^79^
Belgium	0.77	^78^
Bangladesh	0.77	^80^
Türkiye	0.09	^52^

**Fig. 7:**
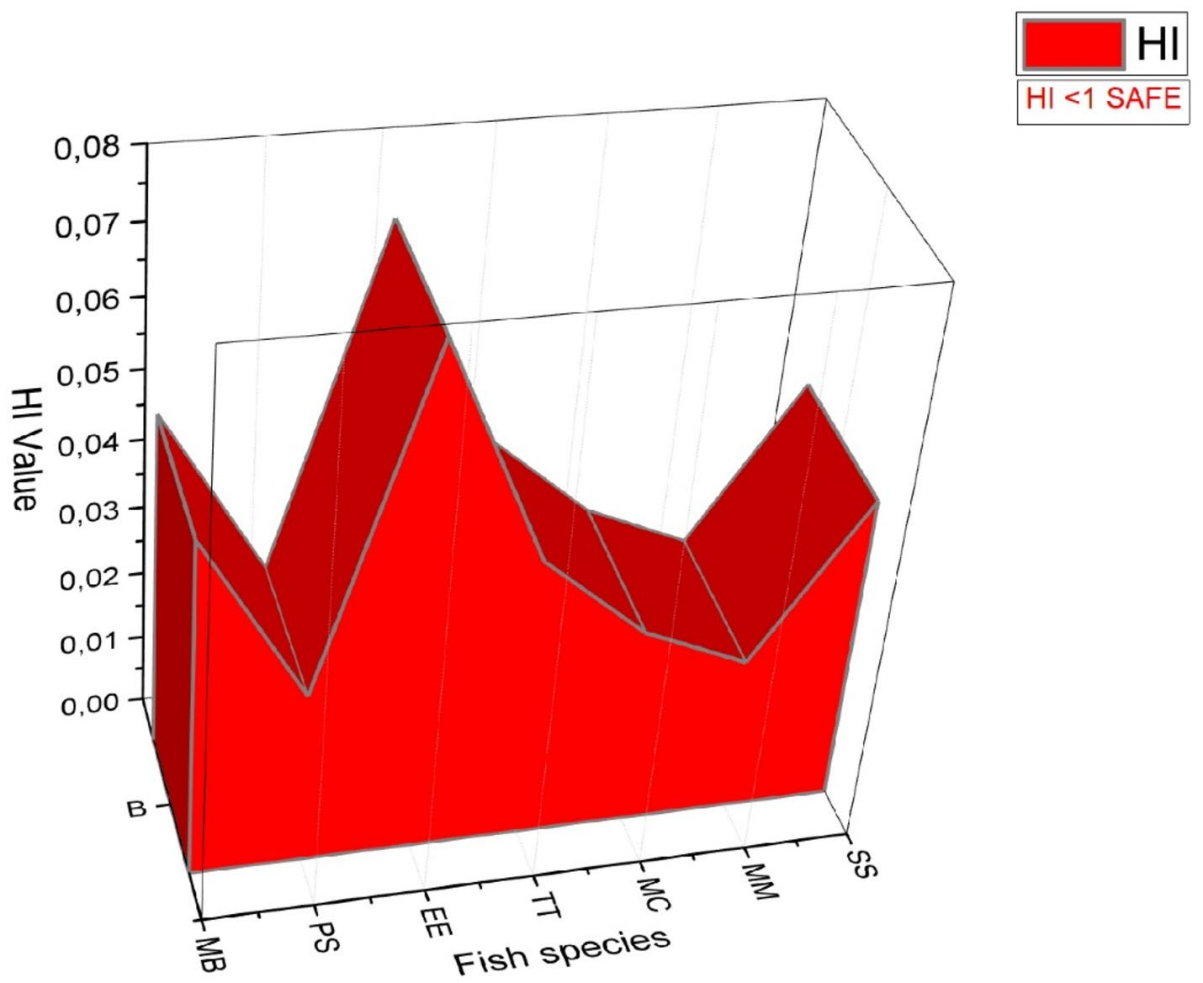
HI calculated for the fish along the Black Sea coastline.

In this study; potential health risk index values were calculated and are shown in Table 4 as THQ, HI and MPI of economic importance seven fish species collected from different cities at the Black Sea coastline. In the calculations, THQ value was determined below 1 for 9 PTEs. Therefore, it can be said that the fish used in the study will not pose an ecotoxic public health risk. Among the seven fish species examined, it was determined that MB, a benthic species, has the highest HI value, while MM has the lowest HI value (Fig. 7). Besides that, seven fish species have Hazard Index (HI) values that are lower than 1 and are considered to not be likely to be harmful to the public’s health (Table 4; Fig. 7).

**Table 4:** Metal pollution index (MPI) for different PTEs, their target hazard quotient (THQ), and hazard index (HI) from consumption of seven fish collected from different cities at the Black Sea coast.

Criteria of Assesment	HTEs	MB	PS	EE	TT	MC	MM	SS
THQ	Pb	2.65E-03	1.32E-03	1.32E-03	6.64E-04	1.99E-03	1.32E-03	6.64E-04
	Ni	1.72E-03	5.31E-04	1.59E-03	5.31E-04	9.30E-04	3.98E-04	2.65E-04
	Cd	7.97E-03	2.65E-03	1.86E-02	7.97E-03	5.31E-03	5.31E-03	5.31E-04
	Al	7.83E-03	1.16E-03	8.26E-04	9.80E-04	1.59E-03	1.33E-03	6.21E-04
	Cr	7.08E-06	1.77E-06	1.77E-06	1.77E-06	7.08E-06	1.77E-06	1.77E-06
	Cu	4.18E-03	5.31E-03	1.08E-02	7.37E-03	5.44E-03	4.11E-03	1.00E-02
	Fe	1.44E-02	3.76E-03	7.45E-03	5.27E-03	5.58E-03	2.83E-03	5.92E-03
	Mn	2.63E-03	6.07E-04	4.08E-03	6.45E-04	6.64E-04	6.07E-04	3.41E-04
	Zn	1.05E-02	9.91E-03	2.84E-02	1.78E-02	7.49E-03	7.74E-03	6.59E-03
HI		4.92E-02	2.52E-02	7.30E-02	4.12E-02	2.90E-02	2.26E-02	4.49E-02
MPI		6.85E-01	2.39E-01	4.57E-01	3.19E-01	3.22E-01	0.273	2.25E-01

*THQ* target hazard quotients,* HI* hazard index,* MPI* metal pollution index.

**Fig. 8:**
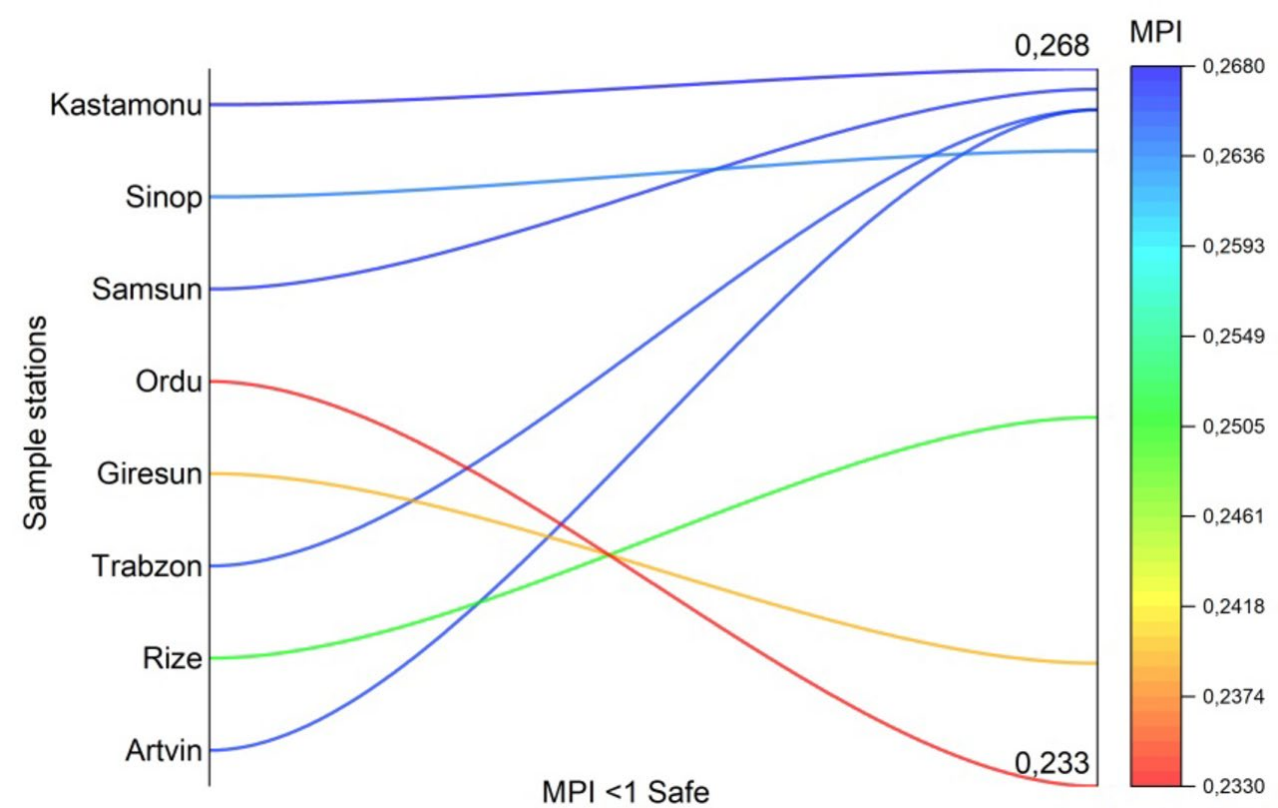
MPI values in the coastal cities of the Black Sea where 7 fish species are fished.

Within the scope of this study, the Metal Pollution Index of seven different fish species caught from eight cities on the Black Sea coast was calculated and shown in Fig. 8. In all cities where fish sampling was carried out, the Metal Pollution Index values of all fish species according to the cities were calculated below 1. As can be seen in Fig. 8, the cities with the highest MPI values are Kastamonu city (0.268 µg g-1), Samsun city (0.267 µg g-1), Artvin city (0.266 µg g-1) and Trabzon city (0.266 µg g-1). It is thought that the intensive harbour activities in Kastamonu, Samsun, Artvin and Trabzon cities cause an increase in heavy metal pollution due to the discharge of streams into the sea from these points, and therefore the MPI level is high for this reason.

Mean daily fish consumption in Türkiye is 20 g per person^54,81^. This is equivalent to 140 g per person per week. In Table 5, the EWI values of a 70 kg adult consuming 140 g of fish per week were estimated, and then estimated daily intake (EDI) values were calculated from the EWI values. The calculated EWI and EDI data are above the MPLs for aluminum in *M. barbatus* at all stations except Ordu and Sinop cities. In *P. saltatrix*, it is above the MPLs only in Trabzon city. Arsenic is above the MPLs only in *M. barbatus* samples from Kastamonu city. In all other fish samples, the calculated data are below the MPLs^53,82,83^. This results, suggesting that the fish species examined in the current research are deemed safe for consumption.

**Table 5:** Comparison of EWI (EDI) with the recommended values for the fish species studied in this study.

Metals	PTWI*	PTWI**	PTDI	M. barbatus EWI (EDI)	*P*. saltatrix EWI (EDI)	E.encrasicolus EWI (EDI)	T. trachurus EWI (EDI)	M. cephalus EWI (EDI)	M. merlangus EWI (EDI)	S. sarda EWI (EDI)	Türkmen and Öğütçü^12^	Erdem et al.,^47^
Al	28.6	2.002	286	7.888 (1.127)	2.487 (355)	1.597 (228)	1.362 (195)	1.512 (216)	1.664 (238)	1.226 (175)	–	–
B	–	–	–	282 (40.25)	177 (25.25)	249 (35.59)	281 (40.16)	447 (63.88)	320 (45.67)	212 (30.28)	–	–
Cu	3.500a	245.000	35.000	112 (15.96)	150 (21.38)	291 (41.54)	206 (29.41)	242 (34.59)	101 (14.43)	475 (67.83)	165 (23.6)	1.98 (0.28)
Fe	5.600a	392.000	5.6000	10.324(1.475)	1.784 (255)	3.315 (474)	2.631 (376)	3.620 (517)	1300 (186)	3.863 (552)	–	27.15 (3.88)
Mn	980b	68.600	9.800d	286 (40.87)	73.79 (10.54)	481 (68.78)	78.55 (11.22)	88.12 (12.59)	62.77 (8.97)	39.90 (5.70)	617 (88.2)	–
Se	–	–	–	234 (33.42)	188 (26.83)	159 (22.71)	305 (43.51)	342 (48.82)	202 (28.81)	206 (29.42)	–	–
Si	–	–	–	8.411 (1.202)	4.374 (625)	4.883 (698)	4.815 (688)	7.441 (1.063)	5.831 (833)	4.840 (691)	–	–
Zn	7.000a	490.000	70.000	1.898 (271)	1.821 (260)	5.621 (803)	3.469 (496)	2.077 (297)	1.489 (213)	1.198 (171)	2366 (338)	46.52 (6.65)
As	1500	1050	150	1.548 (221)	239 (34.20)	418 (59.72)	308 (43.99)	209 (29.87)	241 (34.49)	287 (40.96)	–	3.29 (0.47)
Cd	7a	490	70	8.51 (1.22)	2.83 (0.40)	12.05 (1.72)	9.25 (1.32)	6.33 (0.90)	3.39 (0.48)	1.24 (0.18)	14 (2)	0.03 (0.04)
Co	–	–	–	17.60 (2.51)	1.77 (0.25)	15.55 (2.22)	4.70 (0.67)	6.61 (0.94)	3.64 (0.52)	1.82 (0.26)	22.4 (3.2)	–
Cr	23.3	1.631	233	12.94 (1.85)	4.50 (0.64)	1.91 (0.27)	4.16 (0.59)	14.63 (2.09)	3.65 (0.52)	3.78 (0.54)	322 (46)	0.06 (0.009)
Hg	4	280	40	32.62 (4.66)	5.26 (0.75)	2.25 (0.32)	8.83 (1.26)	3.37 (0.48)	7.29 (1.04)	7.48 (1.07)	–	–
Ni	35b	2.450	350c	31.69 (4.53)	15.56 (2.22)	22.68 (3.24)	15.97 (2.28)	15.79 (2.26)	5.75 (0.82)	6.33 (0.90)	3360 (480)	
Pb	25a	1.750	250	7.83 (1.12)	2.78 (0.40)	3.95 (0.56)	2.58 (0.37)	10.96 (1.57)	6.05 (0.86)	2.12 (0.30)	79.8 (11.4)	0.15 (0.02)

Average weekly fish consumption in Türkiye is 0.14 kg per person^84^. PTWI*, Temporary Tolerable weekly intake (µg/week/kg body weight). PTWI** Temporary Tolerable weekly intake (µg/week/70 kg body weight). EWI, Estimation of Weekly Intake Rate (µg/week/70 kg body weight). ^a^FAO/WHO^82^. ^b^ Calculated for one week (µg/week/kg body weight). ^c^WHO^85^ 5 µg/day/kg a body weight worth TDI(tolerable daily intake), i.e. 350 µg/day for a 70 kg person^85^. ^d^EPA, 0,14 mg/day/kg body weight RfD (reference dose) proposes, i.e. 9800 mg/day for a 70 kg person^83^.

### Correlation between heavy metals in the fishes samples

**Fig. 9:**
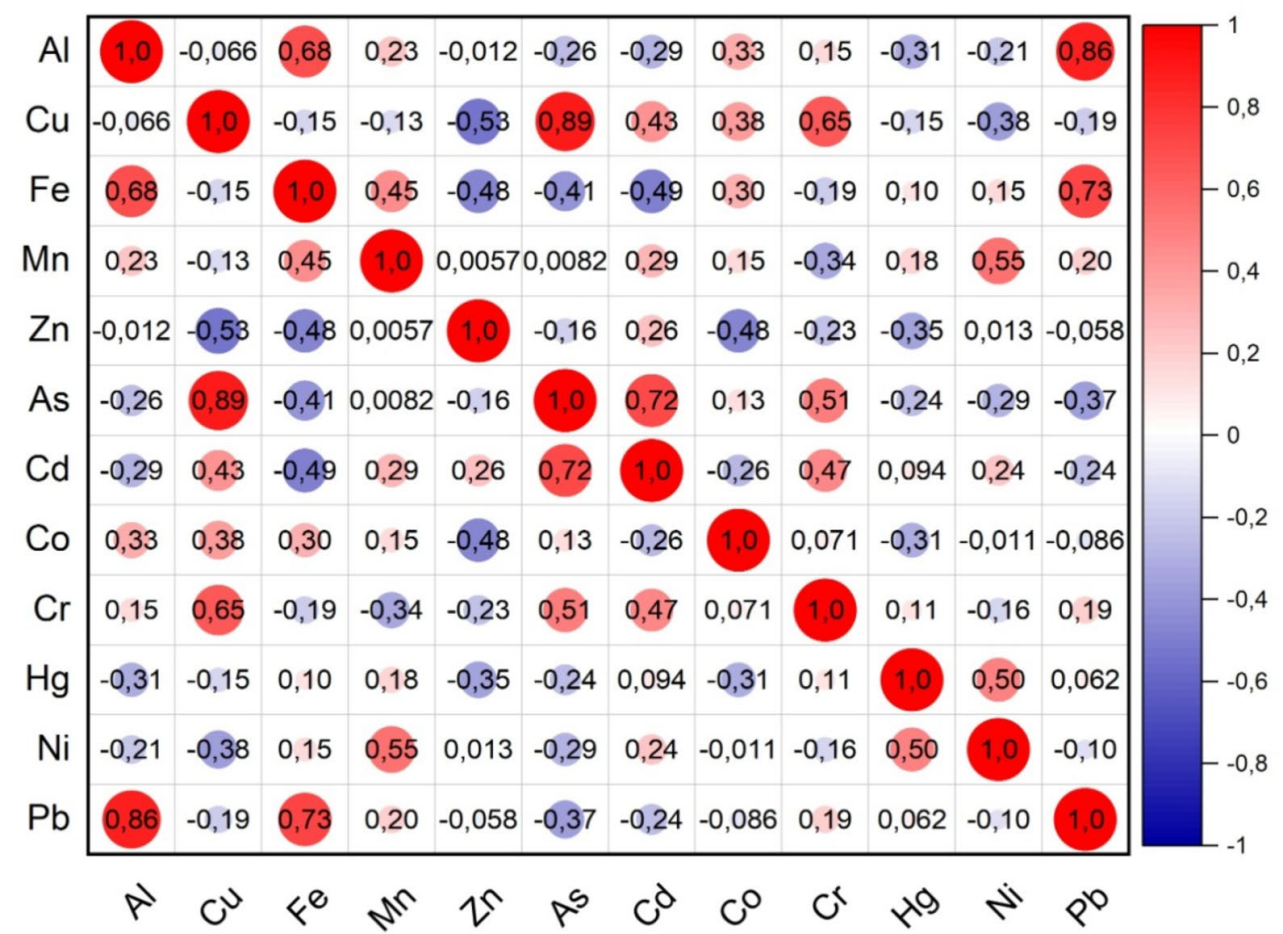
Correlation of PTEs in fish in the Black Sea.

Pearson correlation matrix was utilised in the present study to determine the significant relationships between the sources of PTEs contents and the distribution patterns of the studied fish in the provinces. Positive correlations between metal contents indicate that anthropogenic activities are a common source of these elements. For example, similarities in the concentrations of two different metals identify related sources, the same properties and dependence on the aquatic environment^80^. Pearson correlation coefficients for PTEs in fish sampled in 8 provinces are given in Fig. 9. For fish samples, As and Cu (*r* = 0.89), Pb and Al (0.86), As and Cd (*r* = 0.72), Pb and Fe (*r* = 0.73) were highly correlated. Al, Fe and Pb were found to have a high correlation with each other. Al and Fe are the two highest elements found due to the geogenic structure of the region. For this reason, fishes naturally absorb these elements in large amounts from the rocks into the sea. The presence of arsenic is thought to be of lithogenic and anthropogenic origin.

### Two-way cluster analysis

**Fig. 10:**
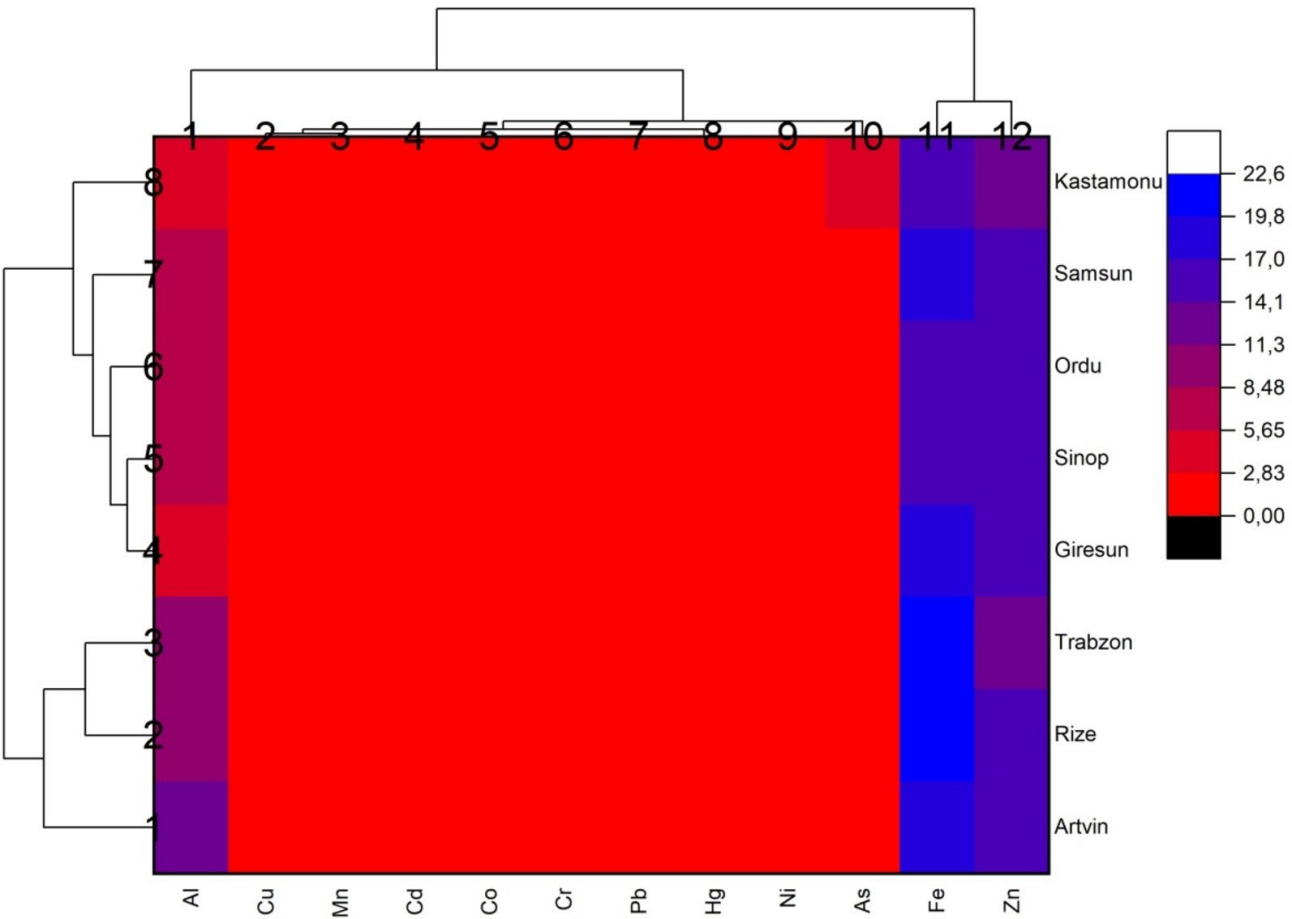
Two-way hierarchical cluster heat map of the provinces where fish sampling was conducted and the level of PTEs.

In the present study, a cluster heat map and dendrogram of the sampled provinces were generated by Ward linkage approach using Euclidean distance (Fig. 10). By creating a cluster of similar species, various species are placed in separate clusters, which helps to identify species with the same concentration of PTEs and the degree of pollution in the sampled species in the provinces^86^. As seen in Fig. 10, the Dendrogram presented two clusters in the vertical section: Cluster 1 included the metals Al, Cu, Mn, Cd, Co, Cr, Pb, Hg, Ni, and As, and Cluster 2 included Fe and Zn, which is consistent with the results of the thematic spatial analysis map of PTEs generated by GIS showing the distribution of bioaccumulation of the selected metals in the cities. Among all analysed provinces, Kastamonu, Samsun, Ordu, Sinop and Giresun were assigned to cluster 1. Cluster 2 included only Trabzon, Rize and Artvin provinces (Fig. 10). It was found that the results of the thematic spatial analysis map of PTEs generated by two-way cluster analysis and GIS were compatible with each other and there was a high correlation between PTEs.

## Conclusion

According to the results of this study; there are different level of PTEs in the muscle of seven fish species and the amount of accumulation varies depending on the species and habitat of the fish. The average PTEs levels in the seven fish muscle are listed in the following order: Fe > Zn > As > Cu > Mn > Ni > Co > Hg > Cd > Pb > Cr with the values of 17.028 > 14.288 > 2.045 > 1.017 > 0.721 > 0.064 > 0.032 > 0.031 > 0.027 > 0.021 > 0.018 µg g^− 1^, respectively. The highest level of mean potentially toxic elements concentration was found in EE (58.16 µg g^− 1^) and MB (57.66 µg g^− 1^ ), while the lowest level was found in MM (18.75 µg g^− 1^). The maximum values of the PTEs measured in fish muscle tissue were mostly observed in Kastamonu, Sinop, Artvin and Rize provinces. In addition, maximum values of Co, Fe, Ni, Si, Al, As, Hg were found in MB and maximum values of B, Cr, Se, Pb were found in MC. The reason for the different metal accumulation in MB and MC fishes compared to other fish species is thought to be due to the feeding habits of MB and MC as well as the fact that MB is a bottom-dwelling species. The differences between the stations for all elements evaluated in the study are statistically significant (*p* < 0.05). B and Fe for MB, Cu for TT, B, Ni and Al for MC, Cu, Fe and As for SS are different in each station. This difference is statistically significant (*p* < 0.05). According to the study, it was observed that heavy metal concentrations were mostly higher in small fish. The negative relationship between metal concentrations and fish size can be explained by the fact that small fish accumulate more metals in their bodies due to their faster metabolism, their immune systems are not developed and they are more active in feeding. When examining the correlation between the metals measured in fish samples, the highest values were found between As and Cu (*r* = 0.89), as well as between Pb and Al (0.86). High correlation values indicate that the accumulation mechanisms of these elements in living organisms are similar and that they enrich each other. The cities with the highest MPI level are the cities of Kastamonu (0.268 µg g-1), Samsun (0.267 µg g-1), Artvin (0.266 µg g-1) and Trabzon (0.266 µg g-1), respectively. For all PTEs, the metal pollution index (MPI), target hazard quotients (THQ) and hazard index (HI) from metal intake by ingesting seven fish species were less than 1, indicating no risk from consumption. The EWI and EDI values are well below the recommended values according to FAO/WHO^82^; WHO^85^; USEPA^83^. The predicted daily intakes of PTEs in each fish species were far lower than their corresponding acceptable daily intakes, indicating that consuming fish would not put consumers at risk for probably cancer problems from daily intakes of PTEs. It should be noted that trace elements like Cu and Zn, despite participating in metabolic activities, can easily exhibit toxic effects with increased doses due to bioaccumulation, while heavy metals like As and Cd can show toxic effects even at low doses. Even if the values obtained in the study are low, continuous mandatory monitoring efforts are necessary. In the Black Sea, especially in industrial and port areas, marine species may pose a risk to public health, so it is recommended that they are not continuously consumed by people.

## Electronic supplementary material

Below is the link to the electronic supplementary material.

Supplementary Material 1

## Data Availability

“Data is provided within the supplementary information files”.
