# Supplementary material for: Ecotoxicological health risk analysis of different fish species from the Black Sea coast of Türkiye
Source: Sci Rep. 2025 Aug 22;15:30883. doi: 10.1038/s41598-025-87875-y (PMC12373849; doi:10.1038/s41598-025-87875-y)
Supplement: Supplementary file 1 — Supplementary Material 1 [file 41598_2025_87875_MOESM1_ESM.docx]

**Supplementary Material**

**Effect of potentially toxic element accumulation on public health: Ecotoxicological health risk analysis of seven different fish along the Black Sea coastline in Türkiye by geographical information systems**

Evrim Sibel ÖNEL^a^, Mustafa TÜRKMEN^a^, Erkan KALIPCI^b,*^

^a^ *Giresun University, Faculty of Science, Department of Biology, Giresun city, Türkiye*

^b^ *Giresun University, Faculty of Engineering, Deparment of Geomatics Engineering, Giresun city, Türkiye*

**Corresponding author: erkan.kalipci@giresun.edu.tr*

**1.Analysis of Health Risk Indexes**

**1.1. Assessment of Metal Pollution Index (MPI)**

The Metal Pollution Index is a mathematical model that epitomize the value for all metals in a single form. MPI is a credible and accurate index to monitor PTEs contamination in the food and the aquatic ecosystem. In this study; MPI was calculated following the equation proposed by Usero et al. (1997):

MPI = (Cf_1_xCf_2_xCf_3_x … xCf_n_)^1/n^ (1)

where: Cf_i_: is the mean concentration of metal i in the samples, n: total number of metals.

**1.2. Assessment of Target Hazard Quotient (THQ)**

THQ is an estimation of the risk level (non-carcinogenic) due to pollutant exposure. The method used to estimate the THQ of each heavy metal is calculated with the formula below (Chien et al., 2002):

$$THQ=\frac{E_{fr}\times{ED}_{tot}\times FIR\times C}{{RfD}_{o}\times{BW}_{a}\times{AT}_{n}}\times{10}^{-3} (2)$$

Efr: Frequency of exposure (365 days year^−1^), EDtot: Period of exposure (average life expectancy:30 years), FIR: Food intake rate (18.6 g day^−1^), C: Mean heavy metal concentration in fish muscular tissue (µg g^-1^ ww), *RfDo: Oral reference dose (µg g^-1^ ww), BWa: Average body weight (70 kg of body weight refers to adult people), ATn: Period of average exposure for non-carginogens (365 days year^−1^ × number of exposure years).

THQ should not exceed 1, else it indicates to pose potential noncarcinogenic risks to exposed population. The health risk assessments are based on assumptions that for most chemicals with noncancer effects, exhibit a threshold response (Ahmed et al., 2016). The acceptable guideline value for THQ is 1 (USEPA, 2011).This method of risk estimation has recently been used by many researchers and has been shown to be valid and useful (Mahmoud and Abdel-Mohsein, 2015; Töre et al., 2021; Varol et al, 2019).

**1.3.** **Assessment of Hazard Index (HI)**

The HI is the summation of the THQ for all metals observed (Li et al., 2013):

$$HI=\sum_{i=1}^{n} {THQ}_{i} (3)$$

Where HI< 1 is safe, HI> 1 is hazardous

When HI<1 then it means the health benefit of fish consumption and that the consumers is safe, whereas HI>1 suggested a high adverse health risks.

**1.4.** **Assessment of Estimation of Weekly Intake Rate (EWI) and Estimation of Daily Intake Rate (EDI)**

The daily and weekly intakes were estimated for fish species examined in this study. The method used to estimate the daily and weekly intakes of each heavy metal is calculated with the formula below (Türkmen et al., 2010):

[EWI (micrograms per 70 kg body weight per week) = Mean metal levels (micrograms per kilogram) × fish consumption (kilogram per 70 kg body weight per week)]. (4)

The average daily fish consumption in Türkiye is 20 g per person (FAO, 2008). Then, EDI values were calculated from EWI values.

**Table SM1.** Comparison of mean PTEs concentrations detected in *M. barbatus* fish muscle tissues with other studies and MPLs for fish (µg g^-1^ ww)

|  | **Al** | **As** | **B** | **Cd** | **Co** | **Cr** | **Cu** | **Fe** | **Hg** | **Mn** | **Ni** | **Pb** | **Se** | **Zn** | **Reference** |
| --- | --- | --- | --- | --- | --- | --- | --- | --- | --- | --- | --- | --- | --- | --- | --- |
|  | – | – | – | 0.05 | – | – | – | – | 0.50 | – | – | 0.30 | – | – | (TFC, 2009) |
|  | – | – | – | 0.05 | – | – | – | – | 0.50 | – | – | 0.30 | – | – | (EC, 2006) |
|  | – | – | – | 1 | – | 50 | 30 | 100 | – | 1 | 0.50–1 | 2 | – | 100 | (WHO, 2014) |
|  | 0.10 | 0.10 | – | 0.10 | – | 2 | – | – | – | – | – | 0.50 | – | – | (MHPRC, 2013) |
|  | – | – | – | 0.50 | – | – | 30 | – | 0.50 | – | – | 0.50 | – | 30 | (FAO, 1983) |
|  | – | 0.60 | – | – | – | 0.03 | – | – | – | – | 0.05 | 0.30 | – | – | (ISNO, 2012) |
|  | **29.50** | **5.31** | **1.48** | **0.03** | **0.08** | **0.04** | **0.63** | **37.96** | **0.09** | **1.39** | **0.13** | **0.04** | **1.22** | **11.96** | **The mean concentrations of this study*** |
| **Sampling station** | **Al** | **As** | **B** | **Cd** | **Co** | **Cr** | **Cu** | **Fe** | **Hg** | **Mn** | **Ni** | **Pb** | **Se** | **Zn** | **Reference** |
| TRB | – | – | – | 0.02 | 0.11 | 0.17 | 1.30 | 27.7 | – | 0.38 | 1.24 | 0.22 | – | 8.26 | (Tepe et al., 2008) |
| SNP | – | – | – | 0.03 | 0.07 | 0.15 | 0.87 | 21.8 | – | 0.48 | 1.05 | 0.39 | – | 10.5 |  |
| BRT | – | – | – | 0.02 | 0.03 | 0.24 | 0.15 | 10.8 | – | 0.28 | 0.20 | 0.17 | – | 5.06 |  |
| SMS | – | 1.33 | – | <0.02 | – | – | – | – | <0.05 | – | – | 0.08 | – | – | (Daş et al., 2009) |
| SNP | – | 2.38 | – | <0.02 | – | – | – | – | <0.05 | – | – | 0.05 | – | – |  |
| BRT | 2.60 | – | 6.73 | 0.11 | – | 0.14 | 4.08 | 21.20 |  | 0.77 | 0.63 | 1.11 | – | 16.03 | (Fındık and Çiçek, 2011) |
| KRD | – | 3.40 | – | 3.38 | – | – | – | 4.00 | – | 0.13 | <0.01 | <0.01 | 0.55 | – | (Aydın and Tokalıoğlu, 2014) |
| SNP | <0.5 | 1.30 | – | <0.02 | – | – | <0.5 | 2.3 | <0.05 | – | – | <0.05 | – | 3.2 | (Bat et al., 2015) |
| SMS | – | – | – | 0.20 | <0.01 | 0.21 | 1.27 | 24.9 | – | 0.19 | 2.21 | 1.76 | – | 4.95 | (Türkmen and Dura, 2016) |
| SNP | – | – | – | 0.07 | 0.02 | 0.40 | 2.38 | 2.11 | – | 0.32 | 0.21 | 2.94 | – | 9.49 |  |
| KCL | – | – | – | 0.06 | 0.02 | 0.03 | 1.40 | 10.4 | – | 0.11 | 2.85 | 0.88 | – | 5.71 |  |
| RZE | – | – | – | 0.09 | 0.06 | 0.11 | 1.81 | 30.5 | – | 0.32 | 1.78 | 1.30 | – | 5.00 | (Türkmen and Akaydın, 2017) |
| TRB | – | – | – | 0.12 | 0.01 | 0.35 | 1.74 | 49.5 | – | 0.43 | 1.73 | 1.03 | – | 7.15 |  |
| GRS | – | – | – | 0.04 | 0.03 | 0.09 | 1.99 | 44.7 | – | 0.24 | 2.54 | 0.45 | – | 6.02 |  |
| SKR | – | 3.37 – 5.87 | – | 0.02 – 0.05 | – | – | 1.36 – 11.85 | – | 0.01 – 0.03 | – | – | 0.03 – 1.70 | – | 20.80 – 34.94 | (Mol et al., 2017) |
| KRD | 0.54 | 50.34 | – | <0.01 | 0.01 | 0.03 | 0.20 | 2.68 | – | 0.18 | 0.25 | 0.07 | – | 3.03 | (Varol et al., 2019) |
| KRD | – | – | – | 0.02 | – | – | 0.29 | – | 0.04 | – | – | 0.05 | – | 6.40 | (Bat et al., 2020) |
| KRD | – | – | – | 0.08 – 0.10 | 0.03 – 0.10 | 0.49 – 0.74 | 0.32 – 0.39 | – | – | 0.41 – 4.41 | 1.24 – 5.76 | 0.13 – 0.18 | – | 7.92 – 8.55 | (Türkmen and Öğütçü, 2020) |
| KRD | <0.01 – 3.55 | – | – | 0.14 – 0.15 | – | 0.15 – 0.43 | 0.82 – 1.77 | 6.81 – 9.21 | – | 0.74 – 1.63 | – | 0.59 – 0.82 | – | 4.53 – 11.60 | (Mutlu, 2021) |
| KRD | – | 5.83 | – | 0.04 | 0.11 | 0.12 | 0.82 | 62.98 | 0.11 | 2.39 | 0.19 | 0.13 | – | 15.48 | (Kalıpcı et al., 2023) |

* These are the average values of the data obtained in the present study, regardless of the sampling station.

**Table SM2.** Comparison of mean PTEs concentrations detected in *P. saltatrix* fish muscle tissues with other studies and MPLs for fish (µg g^-1^ ww)

|  | **Al** | **As** | **B** | **Cd** | **Co** | **Cr** | **Cu** | **Fe** | **Hg** | **Mn** | **Ni** | **Pb** | **Se** | **Zn** | **Reference** |
| --- | --- | --- | --- | --- | --- | --- | --- | --- | --- | --- | --- | --- | --- | --- | --- |
|  | – | – | – | 0.05 | – | – | – | – | 0.50 | – | – | 0.30 | – | – | (TFC, 2009) |
|  | – | – | – | 0.05 | – | – | – | – | 0.50 | – | – | 0.30 | – | – | (EC, 2006) |
|  | – | – | – | 1 | – | 50 | 30 | 100 | – | 1 | 0.50–1 | 2 | – | 100 | (WHO, 2014) |
|  | 0.10 | 0.10 | – | 0.10 | – | 2 | – | – | – | – | – | 0.50 | – | – | (MHPRC, 2013) |
|  | – | – | – | 0.50 | – | – | 30 | – | 0.50 | – | – | 0.50 | – | 30 | (FAO, 1983) |
|  | – | 0.60 | – | – | – | 0.03 | – | – | – | – | 0.05 | 0.30 | – | – | (ISNO, 2012) |
|  | **4.40** | **1.06** | **0.95** | **0.01** | **0.01** | **0.01** | **0.80** | **9.93** | **0.02** | **0.32** | **0.04** | **0.02** | **1.04** | **11.19** | **The mean concentrations of this study*** |
| **Sampling station** | **Al** | **As** | **B** | **Cd** | **Co** | **Cr** | **Cu** | **Fe** | **Hg** | **Mn** | **Ni** | **Pb** | **Se** | **Zn** | **Reference** |
| TRB | – | – | – | 0.01 | 0.03 | 0.21 | 0.52 | 41.2 | – | 0.20 | 0.60 | 0.09 | – | 3.85 | (Türkmen et al., 2009) |
| SNP | – | – | – | 0.03 | 0.21 | 0.18 | 2.97 | 40.3 | – | 0.80 | 0.57 | 0.36 | – | 15.9 |  |
| BRT | – | – | – | 0.02 | 0.03 | 0.07 | 1.58 | 33.4 | – | 0.31 | 0.44 | 0.11 | – | 6.93 |  |
| SMS | – | – | – | 0.19 | 0.04 | 0.18 | 1.73 | 30.6 | – | 0.15 | 2.45 | 1.14 | – | 6.83 | (Türkmen and Dura, 2016) |
| SNP | – | – | – | 0.25 | 0.03 | 0.36 | 1.47 | 20.7 | – | 0.07 | 1.61 | 0.78 | – | 5.66 |  |
| KCL | – | – | – | 0.14 | 0.04 | 0.37 | 1.48 | 24.7 | – | 0.06 | 0.40 | 0.49 | – | 6.02 |  |
| SNP | – | – | – | – | – | – | 0.38 | 19.54 | – | – | – | – | – | 15.00 | (Bat et al., 2017) |
| RZ | – | – | – | 0.05 | 0.04 | 0.39 | 2.52 | 43.4 | – | 0.13 | 0.27 | 0.18 | – | 5.75 | (Türkmen and Akaydın, 2017) |
| TRB | – | – | – | 0.04 | 0.05 | 0.51 | 2.24 | 48.0 | – | 0.12 | 0.41 | 0.13 | – | 6.76 |  |
| GRS | – | – | – | 0.20 | 0.03 | 0.28 | 1.89 | 31.4 | – | 0.15 | 1.68 | 0.28 | – | 4.44 |  |
| KRD | – | 0.99 | – | 0.02 | 0.01 | 0.04 | 0.72 | 8.81 | 0.02 | 0.27 | 0.10 | 0.02 | – | 11.12 | (Kalıpcı et al., 2023) |

* These are the average values of the data obtained in the present study, regardless of the sampling station.

**Table SM3.** Comparison of mean PTEs concentrations detected in *E. encrasicolus* fish muscle tissues with other studies and MPLs for fish (µg g^-1^ ww)

|  | **Al** | **As** | **B** | **Cd** | **Co** | **Cr** | **Cu** | **Fe** | **Hg** | **Mn** | **Ni** | **Pb** | **Se** | **Zn** | **Reference** |
| --- | --- | --- | --- | --- | --- | --- | --- | --- | --- | --- | --- | --- | --- | --- | --- |
|  | – | – | – | 0.05 | – | – | – | – | 0.50 | – | – | 0.30 | – | – | (TFC, 2009) |
|  | – | – | – | 0.05 | – | – | – | – | 0.50 | – | – | 0.30 | – | – | (EC, 2006) |
|  | – | – | – | 1 | – | 50 | 30 | 100 | – | 1 | 0.50–1 | 2 | – | 100 | (WHO, 2014) |
|  | 0.10 | 0.10 | – | 0.10 | – | 2 | – | – | – | – | – | 0.50 | – | – | (MHPRC, 2013) |
|  | – | – | – | 0.50 | – | – | 30 | – | 0.50 | – | – | 0.50 | – | 30 | (FAO, 1983) |
|  | – | 0.60 | – | – | – | 0.03 | – | – | – | – | 0.05 | 0.30 | – | – | (ISNO, 2012) |
|  | **3.11** | **2.35** | **1.33** | **0.07** | **0.05** | **0.01** | **1.63** | **19.64** | **0.01** | **2.15** | **0.12** | **0.02** | **0.97** | **32.11** | **The mean concentrations of this study*** |
| **Sampling station** | **Al** | **As** | **B** | **Cd** | **Co** | **Cr** | **Cu** | **Fe** | **Hg** | **Mn** | **Ni** | **Pb** | **Se** | **Zn** | **Reference** |
| KRD | – | – | – | 0.02 – 0.06 | 0.06 – 0.08 | 0.09 – 0.17 | 0.88 – 8.58 | 35.7 – 44.4 | – | 0.70 – 2.82 | 0.51 – 1.51 | 0.12 – 0.87 | – | 10.6 – 45.6 | (Türkmen et al, 2008) |
| BATUM | – | 0.60 – 0.61 | – | ND | – | – | ND | – | ND | – | – | ND | – | 11.7 – 13.0 | (Bat et al, 2014) |
| ORD | – | 0.41 – 0.67 | – | ND | – | – | ND | – | ND | – | – | ND | – | 11.7 – 12.7 |  |
| SMS | – | 0.65 – 0.67 | – | ND | – | – | ND | – | ND | – | – | ND | – | 9.7 – 12.9 |  |
| SNP | – | 0.53 – 0.69 | – | ND | – | – | ND | – | ND | – | – | ND | – | 8.6 – 9.5 |  |
| SMS | – | – | – | 0.06 | 0.04 | 0.15 | 2.04 | 54.1 | – | 0.51 | 0.14 | 4.07 | – | 17.6 | (Türkmen and Dura, 2016) |
| SNP | – | – | – | 0.19 | 0.03 | 0.16 | 2.08 | 52.5 | – | 0.77 | 0.13 | 2.98 | – | 11.4 |  |
| KCL | – | – | – | 0.08 | 0.05 | 0.12 | 2.44 | 54.0 | – | 0.30 | 0.33 | 4.58 | – | 11.5 |  |
| SNP | – | – | – | – | – | – | 0.28 | 9.75 | – | – | – | – | – | 7.87 | (Bat et al, 2017) |
| RZE | – | – | – | 0.31 | 0.03 | 0.17 | 2.42 | 53.1 | – | 0.63 | 0.54 | 2.99 | – | 11.4 | (Türkmen andAkaydın, 2017) |
| TRB | – | – | – | 0.04 | 0.04 | 0.12 | 2.21 | 52.9 | – | 0.57 | 0.48 | 3.85 | – | 15.4 |  |
| GRS | – | – | – | 0.15 | 0.03 | 0.17 | 2.31 | 53.6 | – | 0.68 | 1.04 | 4.87 | – | 17.6 |  |
| KRD | 1.23 | 3.47 | – | <0.01 | 0.08 | 0.08 | 0.90 | 21.36 | – | 2.02 | 0.11 | 0.02 | – | 39.52 | (Varol et al, 2019) |
| KRD | – | – | – | 0.09 – 0.10 | 0.03 – 0.16 | 0.55 – 2.30 | 0.55 – 1.18 | – | – | 0.59 – 1.13 | 1.62 – 21.2 | 0.19 – 0.33 | – | 14.1 – 16.9 | (Türkmen and Öğütçü, 2020) |
| SMS, SNP | 2.27 | 2.17 | – | 0.02 | 0.04 | 0.04 | 1.31 | 17.93 | 0.14 | 1.55 | 0.65 | 0.10 | 0.63 | 30.72 | (Erdem et al, 2021) |
| KRD | 0.03 – 12.39 | – | – | 0.08 – 0.16 | – | 0.14 – 0.45 | 1.42 – 3.25 | 9.95 – 23.49 | – | 1.06 – 3.24 | – | 0.73 – 1.14 | – | 12.89 – 25.75 | (Mutlu, 2021) |
| KRD | – | 1.95 | – | 0.06 | 0.05 | 0.01 | 1.32 | 17.43 | 0.01 | 1.62 | 0.11 | 0.02 | – | 29.12 | (Kalıpcı et al, 2023) |

* These are the average values of the data obtained in the present study, regardless of the sampling station.

**Table SM4.** Comparison of mean PTEs concentrations detected in *T. trachurus* fish muscle tissues with other studies and MPLs for fish (µg g^-1^ ww)

|  | **Al** | **As** | **B** | **Cd** | **Co** | **Cr** | **Cu** | **Fe** | **Hg** | **Mn** | **Ni** | **Pb** | **Se** | **Zn** | **Reference** |
| --- | --- | --- | --- | --- | --- | --- | --- | --- | --- | --- | --- | --- | --- | --- | --- |
|  | – | – | – | 0.05 | – | – | – | – | 0.50 | – | – | 0.30 | – | – | (TFC, 2009) |
|  | – | – | – | 0.05 | – | – | – | – | 0.50 | – | – | 0.30 | – | – | (EC, 2006) |
|  | – | – | – | 1 | – | 50 | 30 | 100 | – | 1 | 0.50–1 | 2 | – | 100 | (WHO, 2014) |
|  | 0.10 | 0.10 | – | 0.10 | – | 2 | – | – | – | – | – | 0.50 | – | – | (MHPRC, 2013) |
|  | – | – | – | 0.50 | – | – | 30 | – | 0.50 | – | – | 0.50 | – | 30 | (FAO, 1983) |
|  | – | 0.60 | – | – | – | 0.03 | – | – | – | – | 0.05 | 0.30 | – | – | (ISNO, 2012) |
|  | **3.69** | **1.74** | **1.51** | **0.03** | **0.03** | **0.01** | **1.11** | **13.89** | **0.02** | **0.34** | **0.04** | **0.01** | **1.57** | **20.11** | **The mean concentrations of this study*** |
| **Sampling station** | **Al** | **As** | **B** | **Cd** | **Co** | **Cr** | **Cu** | **Fe** | **Hg** | **Mn** | **Ni** | **Pb** | **Se** | **Zn** | **Reference** |
| SNP | – | – | – | 0.04 – 0.05 | – | – | 2.22 – 6.21 | – | – | – | – | 0.17 – 0.23 | – | 17.89 – 32.38 | (Bat et al, 2012) |
| KRD | – | – | – | 0.03 – 0.08 | 0.07 – 0.08 | – | 0.82 – 1.17 | 24.9 – 28.0 | – | 0.39 – 0.86 | 0.27 – 0.83 | 0.16 – 0.41 | – | 4.34 – 8.78 | (Mutlu et al, 2012) |
| KRD | – | 0.63 | – | 3.58 | – | – | – | 3.86 | – | 0.44 | 0.03 | <0.01 | 0.39 | – | (Aydın and Tokalıoğlu, 2014) |
| SMS | – | – | – | 0.11 | 0.04 | 0.26 | 2.11 | 2.09 | – | 0.35 | 0.22 | 1.02 | – | 6.90 | (Türkmen and Dura, 2016) |
| SNP | – | – | – | 0.13 | 0.05 | 0.28 | 2.23 | 2.88 | – | 0.22 | 0.34 | 1.50 | – | 11.5 |  |
| KCL | – | – | – | 0.11 | 0.01 | 0.07 | 1.51 | 39.5 | – | 0.27 | 2.92 | 3.63 | – | 5.70 |  |
| RZ | – | – | – | 0.15 | 0.02 | 0.19 | 1.85 | 40.9 | – | 0.23 | 1.78 | 2.49 | – | 5.57 | (Türkmen andAkaydın, 2017) |
| TRB | – | – | – | 0.16 | 0.02 | 0.43 | 2.41 | 45.7 | – | 0.27 | 1.77 | 0.76 | – | 6.38 |  |
| GRS | – | – | – | 0.76 | 0.04 | 0.09 | 0.28 | 39.4 | – | 0.44 | 1.41 | 1.81 | – | 3.75 |  |
| KRD | 1.63 | 0.68 | – | <0.01 | 0.01 | 0.08 | 0.53 | 5.64 | 0.02 | 0.15 | 0.04 | 0.02 | 0.58 | 7.0 | (Duyar and Bilgin, 2019) |
| KRD | 0.56 | 1.41 | – | <0.01 | 0.10 | 0.03 | 0.74 | 10.61 | – | 0.33 | 0.04 | 0.02 | – | 39.28 | (Varol et al., 2019) |
| KRD | – | – | – | 0.09 | 0.02 – 0.03 | 0.52 – 0.63 | 0.54 – 0.63 | – | – | 0.17 – 0.28 | 1.08 – 5.10 | 0.13 – 0.55 | – | 6.66 – 8.57 | (Türkmen and Öğütçü, 2020) |
| KRD | 0.02 – 3.79 | – | – | 0.12 – 0.04 | – | 0.23 – 0.35 | 0.75 – 2.73 | 7.30 – 14.53 | – | 0.45 – 1.03 | – | 0.82 – 1.34 | – | 5.72 – 32.37 | (Mutlu, 2021) |

* These are the average values of the data obtained in the present study, regardless of the sampling station.

**Table SM5.** Comparison of mean PTEs concentrations detected in *M. cephalus* fish muscle tissues with other studies and MPLs for fish (µg g^-1^ ww)

|  | **Al** | **As** | **B** | **Cd** | **Co** | **Cr** | **Cu** | **Fe** | **Hg** | **Mn** | **Ni** | **Pb** | **Se** | **Zn** | **Reference** |
| --- | --- | --- | --- | --- | --- | --- | --- | --- | --- | --- | --- | --- | --- | --- | --- |
|  | – | – | – | 0.05 | – | – | – | – | 0.50 | – | – | 0.30 | – | – | (TFC, 2009) |
|  | – | – | – | 0.05 | – | – | – | – | 0.50 | – | – | 0.30 | – | – | (EC, 2006) |
|  | – | – | – | 1 | – | 50 | 30 | 100 | – | 1 | 0.50–1 | 2 | – | 100 | (WHO, 2014) |
|  | 0.10 | 0.10 | – | 0.10 | – | 2 | – | – | – | – | – | 0.50 | – | – | (MHPRC, 2013) |
|  | – | – | – | 0.50 | – | – | 30 | – | 0.50 | – | – | 0.50 | – | 30 | (FAO, 1983) |
|  | – | 0.60 | – | – | – | 0.03 | – | – | – | – | 0.05 | 0.30 | – | – | (ISNO, 2012) |
|  | **5.31** | **0.99** | **1.40** | **0.02** | **0.03** | **0.04** | **0.82** | **14.71** | **0.01** | **0.35** | **0.07** | **0.03** | **1.01** | **8.46** | **The mean concentrations of this study*** |
| **Sampling station** | **Al** | **As** | **B** | **Cd** | **Co** | **Cr** | **Cu** | **Fe** | **Hg** | **Mn** | **Ni** | **Pb** | **Se** | **Zn** | **Reference** |
| SNP | – | – | – | 0.02 – 0.03 | – | – | 2.86 – 4.61 | – | – | – | – | 0.09 – 0.19 | – | 30.88 – 42.65 | (Bat et al., 2012) |
| RZE | – | – | – | 0.30 | 0.32 | 0.09 | 0.95 | 41.1 | – | 0.16 | 0.09 | 0.90 | – | 3.99 | (Türkmen and Akaydın, 2017) |
| GRS | – | – | – | 0.57 | 0.32 | 0.07 | 0.57 | 42.9 | – | 0.06 | 0.64 | 0.02 | – | 4.18 |  |

* These are the average values of the data obtained in the present study, regardless of the sampling station.

**Table SM6.** Comparison of mean PTEs concentrations detected in *M. merlangus* fish muscle tissues with other studies and MPLs for fish (µg g^-1^ ww)

|  | **Al** | **As** | **B** | **Cd** | **Co** | **Cr** | **Cu** | **Fe** | **Hg** | **Mn** | **Ni** | **Pb** | **Se** | **Zn** | **Reference** |
| --- | --- | --- | --- | --- | --- | --- | --- | --- | --- | --- | --- | --- | --- | --- | --- |
|  | – | – | – | 0.05 | – | – | – | – | 0.50 | – | – | 0.30 | – | – | (TFC, 2009) |
|  | – | – | – | 0.05 | – | – | – | – | 0.50 | – | – | 0.30 | – | – | (EC, 2006) |
|  | – | – | – | 1 | – | 50 | 30 | 100 | – | 1 | 0.50–1 | 2 | – | 100 | (WHO, 2014) |
|  | 0.10 | 0.10 | – | 0.10 | – | 2 | – | – | – | – | – | 0.50 | – | – | (MHPRC, 2013) |
|  | – | – | – | 0.50 | – | – | 30 | – | 0.50 | – | – | 0.50 | – | 30 | (FAO, 1983) |
|  | – | 0.60 | – | – | – | 0.03 | – | – | – | – | 0.05 | 0.30 | – | – | (ISNO, 2012) |
|  | **5.04** | **1.48** | **1.55** | **0.02** | **0.02** | **0.01** | **0.62** | **7.46** | **0.03** | **0.32** | **0.03** | **0.02** | **1.21** | **8.74** | **The mean concentrations of this study*** |
| **Sampling station** | **Al** | **As** | **B** | **Cd** | **Co** | **Cr** | **Cu** | **Fe** | **Hg** | **Mn** | **Ni** | **Pb** | **Se** | **Zn** | **Reference** |
| TRB | – | – | – | 0.01 | 0.07 | 0.13 | 0.88 | 48.4 | – | 0.57 | 0.83 | 0.25 | – | 8.62 | (Tepe et al., 2008) |
| SNP | – | – | – | 0.04 | 0.06 | 0.19 | 2.90 | 81.9 | – | 0.69 | 1.95 | 0.46 | – | 12.9 |  |
| BRT | – | – | – | 0.02 | 0.05 | 0.13 | 0.77 | 34.0 | – | 0.42 | 0.67 | 0.18 | – | 5.73 |  |
| SMS | – | 1.09 | – | <0.02 | – | – | – | – | <0.05 | – | – | 0.05 | – | – | (Daş et al., 2009) |
| SNP | – | 0.58 | – | <0.02 | – | – | – | – | <0.05 | – | – | 0.05 | – | – |  |
| BRT | 17.36 | – | 44.8 | 0.40 | 0.92 | – | 8.53 | 83.01 |  | 2.13 | 1.96 | 6.80 | – | 77.99 | (Fındık and Çiçek, 2011) |
| KRD | – | 0.82 | – | 4.05 | – | – | – | 0.69 | – | 0.23 | <0.01 | 0.02 | 0.40 | – | (Aydın and Tokalıoğlu, 2014) |
| SNP | <0.5 | 1.24 | – | <0.02 | – | – | <0.5 | 0.87 | <0.05 | – | – | <0.05 | – | 3.4 | (Bat et al., 2015) |
| SMS | – | – | – | 0.06 | 0.02 | 0.04 | 1.28 | 17.7 | – | 0.14 | 3.05 | 1.41 | – | 5.04 | (Türkmen and Dura, 2016) |
| SNP | – | – | – | 0.05 | 0.03 | 0.18 | 0.92 | 8.34 | – | 0.08 | 0.80 | 0.63 | – | 3.47 |  |
| KCL | – | – | – | 0.06 | 0.02 | 0.03 | 1.46 | 16.4 | – | 0.15 | 2.52 | 0.69 | – | 3.99 |  |
| SKR | – | 1.98 – 7.53 | – | 0.01 – 0.04 | – | – | 1.57 – 3.35 | – | 0.01 – 0.02 | – | – | 0.05 – 1.22 | – | 13.70 – 36.49 | (Mol et al., 2017) |
| RZE | – | – | – | 0.08 | 0.03 | 0.04 | 1.65 | 8.16 | – | 0.18 | 2.44 | 1.29 | – | 4.08 | (Türkmen andAkaydın, 2017) |
| TRB | – | – | – | 0.12 | 0.03 | 0.28 | 1.62 | 45.6 | – | 0.30 | 1.80 | 1.30 | – | 5.65 |  |
| GRS | – | – | – | 0.05 | 0.04 | 0.04 | 2.40 | 32.1 | – | 0.43 | 0.09 | 0.66 | – | 3.77 |  |
| KRD | – | – | – | 0.01 | – | – | 0.20 | – | 0.02 | – | – | 0.07 | – | 9.05 | (Bat et al., 2020) |
| KRD | – | – | – | 0.09 – 0.10 | 0.02 – 0.04 | 0.51 – 0.55 | 0.26 – 0.69 | – |  | 0.21 – 0.45 | 1.60 – 2.53 | 0.16 – 0.57 | – | 6.41 – 9.42 | (Türkmen and Öğütçü, 2020) |
| SNP | – | – | – | 1.07 | – | 1.48 | – | – | ND | – | 1.77 | 5.38 | – | – | (Baki, 2021) |
| KRD | 3.5 – 7.39 | – | – | 0.06 – 0.14 | – | 0.21 – 0.39 | 0.71 – 3.15 | 4.64 – 7.96 | – | 0.37 – 2.31 | – | 0.58 – 1.31 | – | 4.76 – 8.65 | (Mutlu, 2021) |
| KRD | – | 1.44 | – | 0.02 | 0.01 | 0.03 | 0.63 | 7.16 | 0.04 | 0.35 | 0.05 | 0.02 | – | 8.66 | (Kalıpcı et al., 2023) |

* These are the average values of the data obtained in the present study, regardless of the sampling station.

**Table SM7.** Comparison of mean PTEs concentrations detected in *S. sarda* fish muscle tissues with other studies and MPLs for fish (µg g^-1^ ww)

|  | **Al** | **As** | **B** | **Cd** | **Co** | **Cr** | **Cu** | **Fe** | **Hg** | **Mn** | **Ni** | **Pb** | **Se** | **Zn** | **Reference** |
| --- | --- | --- | --- | --- | --- | --- | --- | --- | --- | --- | --- | --- | --- | --- | --- |
|  | – | – | – | 0.05 | – | – | – | – | 0.50 | – | – | 0.30 | – | – | (TFC, 2009) |
|  | – | – | – | 0.05 | – | – | – | – | 0.50 | – | – | 0.30 | – | – | (EC, 2006) |
|  | – | – | – | 1 | – | 50 | 30 | 100 | – | 1 | 0.50–1 | 2 | – | 100 | (WHO, 2014) |
|  | 0.10 | 0.10 | – | 0.10 | – | 2 | – | – | – | – | – | 0.50 | – | – | (MHPRC, 2013) |
|  | – | – | – | 0.50 | – | – | 30 | – | 0.50 | – | – | 0.50 | – | 30 | (FAO, 1983) |
|  | – | 0.60 | – | – | – | 0.03 | – | – | – | – | 0.05 | 0.30 | – | – | (ISNO, 2012) |
|  | **2.34** | **1.39** | **0.85** | **0.01** | **0.01** | **0.01** | **1.51** | **15.61** | **0.04** | **0.18** | **0.02** | **0.01** | **1.26** | **7.45** | **The mean concentrations of this study*** |
| **Sampling station** | **Al** | **As** | **B** | **Cd** | **Co** | **Cr** | **Cu** | **Fe** | **Hg** | **Mn** | **Ni** | **Pb** | **Se** | **Zn** | **Reference** |
| SMS | – | – | – | 0.05 | 0.06 | 0.23 | 2.97 | 39.6 | – | 0.13 | 1.24 | 0.35 | – | 32.1 | (Türkmen and Dura, 2016) |
| SNP | – | – | – | 0.13 | 0.03 | 0.36 | 4.13 | 37.2 | – | 0.34 | 2.89 | 0.29 | – | 7.29 |  |
| KCL | – | – | – | 0.11 | 0.04 | 0.34 | 3.46 | 43.1 | – | 0.27 | 2.44 | 0.25 | – | 5.26 |  |
| RZE | – | – | – | 0.04 | 0.03 | 0.10 | 3.78 | 37.8 | – | 0.60 | 4.46 | 0.29 | – | 12.7 | (Türkmen and Akaydın, 2017) |
| TRB | – | – | – | 0.04 | 0.04 | 0.24 | 2.75 | 47.4 | – | 0.16 | 2.94 | 0.37 | – | 11.3 |  |
| GRS | – | – | – | 0.04 | 0.04 | 0.23 | 2.96 | 40.7 | – | 0.23 | 5.91 | 0.52 | – | 12.7 |  |
| KRD | – | – | – | 0.09 | 0.01 – 0.05 | 0.54 – 0.70 | 0.77 – 1.16 | – | – | 0.13 – 0.23 | 1.07 – 4.75 | 0.13 – 0.14 | – | 6.30 – 8.17 | (Türkmen and Öğütçü, 2020) |
| KRD | 0.02 – 8.72 | – | – | 0.06 – 0.14 | – | 0.20 – 0.28 | 1.57 – 4.08 | 5.86 – 32.82 | – | 0.20 – 0.82 | – | 0.73 – 1.11 | – | 4.63 – 18.34 | (Mutlu, 2021) |
| RZE | 17.94 | – | – | – | – | – | 2.46 | 18.42 | – | 2.14 | – | 0.25 | – | 8.40 | (Verep and Mutlu, 2022) |
| TRB | 29.33 | – | – | – | – | – | 1.34 | 10.41 | – | 2.49 | – | 0.33 | – | 16.56 |  |
| KRD | – | 1.55 | – | <0.01 | <0.01 | 0.03 | 1.54 | 15.72 | 0.04 | 0.25 | 0.04 | 0.01 | – | 8.09 | (Kalıpcı et al., 2023) |

* These are the average values of the data obtained in the present study, regardless of the sampling station.

**References**

Aydın, D., Tokalıoğlu, Ş., (2014). Trace metals in tissues of the six most common fish species in the Black Sea, Turkey. *Food Additives ve Contaminants: Part B: Surveillance*, 1-16.

Ahmed, M. K., Baki, M. A., Kundu, G. K., Islam, M. S., Islam, M. M., & Hossain, M. M. (2016). Human health risks from heavy metals in fish of Buriganga river, Bangladesh. *Springer Plus*, 5(1), 1697.

Bat, L., Öztekin, A., Arıcı, E., Şahin, F., (2020). Health risk assessment: heavy metals in fish from southern Black Sea. *Foods and Raw Materials*, 115-124.

Bat, L., Öztekin, H. C., Üstün, F. (2015). Heavy metal levels in four commercial fishes caught in Sinop coasts of the Black Sea, Turkey. *Turkish Journal of Fisheries and Aquatic Sciences*, *15*(2015), 393-399.

Bat, L., Kaya, Y., Öztekin, H. C., (2014). Heavy metal levels in the Black Sea anchovy (*Engraulis encrasicolus*) as biomonitor and potential risk of human health. *Turkish Journal of Fisheries and Aquatic Sciesnes*, 845-851.

Bat, L., Sezgin, M., Üstün, F., Şahin, F., (2012). Heavy metal concentrations in ten species of fishes cauht in Sinop coastal waters of the Black Sea, Turkey. *Turkish Journal of Fisheries and Aquatic Sciences*, 371-376.

Bat, L., Arıcı, E., Öztekin, A., (2017). Metal levels in commercial pelagic fishes and their contribution to their exposure in Turkish people of the Black Sea. *Journal Fish Res.,* (1): 1-4.

Baki, O. G., (2021). The evaluation of heavy metal accumulation in whiting fish (*Merlanguis merlangus euxinus,* Nordmann, 1840), a local and economis species of the ventral Black Sea region. *Turkish Journal of Agriculture - Food Science and Technology*, 683-688.

Chien LC, Hung TC, Choang KY, et al. (2002). Daily intake of TBT, Cu, Zn, Cd and As for fishermen in Taiwan. *Sci Total Environ* 285:177–185.

Daş, Y.K., Aksoy, A., Başkaya, R., Duyar, H. A., Güvenç, D., Boz, V., (2009). Heavy metal levels of some marine organisms collected in Samsun and Sinop Coast of Black Sea, in Turkey. *Journal of Animal and Veterinary Advences*, 51.

Duyar, H. A., Bilgin, S., (2019). Heavy metal concentrations in different matinr organism obtained from the Black Sea, Turkey. *Fresenius Environmental Bulletin*, (28): 5281-5286.

EC, C. R. (2006). Official journal of the european union. Setting maximum levels for certain contaminants in food stuffs. Commission regulation. (EC)No 1881/2006, 364: 5-24.

Erdem, M. E., Köstekli, B., Keskin, İ., Kocatepe, D., Kaya, Y., (2021). Mineral matter content and heavy metal contamination of anchovy (*Engraulis ancrasicolus,* Linnaeus 1758) captured from different seas. *KSU J. Agr.c Nat.,* 285-292.

FAO (Food and Agriculture Organization) (1983) Compilation of legal limits for hazardous substances in fish and fishery products. FAO Fishery Circular No. 464. Food and Agriculture Organization of the United Nations, Rome.

FAO (2008). Fisheries and aquaculture, Turkey. Resource document. Food and Agriculture Organization of the United Nations. <http://www.fao.org/fishery/countrysector/naso_turkey>

Fındık, Ö., Çiçek, E., (2011). Metal concentrations in two bioindicator fish species, *Merlangius merlangus, Mullus barbatus,* captured from the West Black Sea coasts (Bartin) of Turkey. *Bull. Environ. Contam. Toxicol.,* (87): 399-403.

ISNO, (2012). Iran Standard National Organization. Instructions and regulations for consuming marine contaminated fish species 2 (15).

Kalıpcı, E., Cüce, H., Ustaoğlu, F., Dereli, M. A., Türkmen, M. (2023). Toxicological health risk analysis of hazardous trace elements accumulation in the edible fish species of the Black Sea in Türkiye using multivariate statistical and spatial assessment. *Environmental Toxicology and Pharmacology*, *97*, 104028.

Li, J., Huang, Zh.Y., Hu, Y., Yang, H., (2013). Potential risk assessment of heavy metals by consuming shellfish collected from Xiamen, China. Environ. Sci. Pollut. Res. 20 (5), 2937–2947.

Mahmoud, M. A., Abdel-Mohsein, H. S. (2015). Health risk assessment of heavy metals for Egyptian population via consumption of poultry edibles. *Adv. Anim. Vet. Sci*, *3*(1), 58-70.

Mol, S., Karakulak, F. S., Ulusoy, Ş., (2017). Assessment of potential health risks of heavy metals to the general public in Turkey via consumption of red mullet, whiting, turbot from the Southwest Black Sea. *Turkish Journal of Fisheries and Aquatic Sciences*, 1135-1143.

Mutlu, T., (2021). Heavy metal concentrations in the edible tissues of some commercial fishes caught along the Eastern Black Sea coast of Turkey and the health risk assessment. *Spectroscopy Letters,* 437-445.

Mutlu, C., Türkmen, A., Türkmen, M., Tepe, Y., Ateş, A., (2012). Comparison of the heavy metal concentrations in Atlantic horse mackerel, *Trachurus trachurus*, from coastal waters of Turkey. *Fresenius Enviromental Bulletin, 21(2), 304-307.*

Mutlu, T., (2021). Heavy metal concentrations in the edible tissues of some commercial fishes caught along the Eastern Black Sea coast of Turkey and the health risk assessment. *Spectroscopy Letters,* 437-445.

MHPRC (Ministry of Health of the People’s Republic of China), (2013). National Food Safety Standard, Maximum Levels of Contaminants in Foods (GB2762–2012).

Tepe Y, Turkmen M, Turkmen A. (2008). Assessment of heavy metals in two commercial fish species of four Turkish seas. *Environ Monit Assess*., 146:277–84.

Türkmen, A., Türkmen, M., Tepe, T., Çekiç, M., (2010). Metals in tissues of fish from Yelkoma Lagoon, northeastern Mediterranean. *Environ. Monit. Assess,* (168):223-230.

Türkmen, M., Dura, N., (2016). Assessment of heavy metal concenrtations in fish from south western Black Sea. *Indian Journal of Geo Marine Sciences*, 1552-1559.

Türkmen, M., Öğütçü, B. (2020). Assessment of Heavy Metals in Selected Fish Species from Markets in the Black Sea Region of Turkey. *Journal of Anatolian Environmental and Animal Sciences*, *5*(4), 636-639.

Türkmen, M., Türkmen, A., Tepe, Y., Töre, Y., & Ateş, A. (2009). Determination of metals in fish species from Aegean and Mediterranean seas. *Food chemistry*, 113(1), 233-237.

Türkmen, M., Türkmen, A., Tepe, Y., Ateş, A., Gökkuş, K., (2008). Determination of Metal Contaminations in Sea Foods from Marmara, Aegean and Mediterranean Seas: Twelve Fish Species. *Food Chemistry,* 108: 794-800.

Türkmen, M., Akaydın, A., (2017). Metal levels in tissues of commercially important fish species from Southeastern Black Sea Coasts. *Indian Journal of Geo Marine Sciences*, (11): 2357-2360.

Töre, Y., Ustaoğlu, F., Tepe, Y., Kalipci, E. (2021). Levels of toxic metals in edible fish species of the Tigris River (Turkey); threat to public health. *Ecological Indicators*, *123*, 107361.

TFC, T. F. (2009). Notifications changes to the maximum levels for certain contaminants in foodstuffs (in Turkish). (Notification No:2009/22), iss. 27143.

Usero J, Gonza´lez-Regalado E, Gracia I (1997). Trace metals in the bivalve molluscs Ruditapes decussatus and Ruditapes philippinarum from the atlantic coast of Southern Spain. *Environ Int,* 23:291–298.

USEPA (U.S. Environmental Protection Agency) (2011). Exposure factors handbook 2011 edition (Final). National Center for Environmental Assessment, Office of Research and Development, Washington D.C.

Varol, M., Kaya, G. K., Sünbül, M. R. (2019). Evaluation of health risks from exposure to arsenic and heavy metals through consumption of ten fish species. *Environmental Science and Pollution Research,* *26*(32), 33311-33320.

Verep, B., Mutlu, T., (2022). Heavy metal concentrations and health risk assessment in Sarda sarda (Bloch, 1793) caught in the Turkish Black Sea coasts. *Acta Aquatica Turcica*, 208-216.

WHO. (2014). Guidelines for Drinking Water Quality, 2nd edn, Chemical aspects. (Geneva: WHO). http://www.who.int/water_sanitation_health/dwq/g dwq2v1/en/
